# Malignant Ascites in Ovarian Cancer: Cellular, Acellular, and Biophysical Determinants of Molecular Characteristics and Therapy Response

**DOI:** 10.3390/cancers13174318

**Published:** 2021-08-26

**Authors:** Brittany P. Rickard, Christina Conrad, Aaron J. Sorrin, Mustafa Kemal Ruhi, Jocelyn C. Reader, Stephanie A. Huang, Walfre Franco, Giuliano Scarcelli, William J. Polacheck, Dana M. Roque, Marcela G. del Carmen, Huang-Chiao Huang, Utkan Demirci, Imran Rizvi

**Affiliations:** 1 Curriculum in Toxicology & Environmental Medicine, School of Medicine, University of North Carolina at Chapel Hill, Chapel Hill, NC 27599, USA; brickard@live.unc.edu; 2 Joint Department of Biomedical Engineering, University of North Carolina at Chapel Hill, Chapel Hill, NC, and North Carolina State University, Raleigh, NC 27599, USA; mkruhi@email.unc.edu (M.K.R.); stephhua@live.unc.edu (S.A.H.); polacheck@unc.edu (W.J.P.); 3 Fischell Department of Bioengineering, University of Maryland, College Park, MD 20742, USA; cconrad8@terpmail.umd.edu (C.C.); asorrin@terpmail.umd.edu (A.J.S.); scarc@umd.edu (G.S.); hchuang@umd.edu (H.-C.H.); 4 Department of Obstetrics, Gynecology and Reproductive Medicine, School of Medicine, University of Maryland, Baltimore, MD 21201, USA; jreader@som.umaryland.edu (J.C.R.); droque@som.umaryland.edu (D.M.R.); 5 Marlene and Stewart Greenebaum Cancer Center, School of Medicine, University of Maryland, Baltimore, MD 21201, USA; 6 Department of Biomedical Engineering, University of Massachusetts Lowell, Lowell, MA 01854, USA; walfre_Franco@uml.edu; 7 Department of Cell Biology and Physiology, School of Medicine, University of North Carolina at Chapel Hill, Chapel Hill, NC 27599, USA; 8 Lineberger Comprehensive Cancer Center, School of Medicine, University of North Carolina at Chapel Hill, Chapel Hill, NC 27599, USA; 9 Division of Gynecologic Oncology, Vincent Obstetrics and Gynecology, Massachusetts General Hospital, Harvard Medical School, Boston, MA 02114, USA; mdelcarmen@mgh.harvard.edu; 10 Bio-Acoustic MEMS in Medicine (BAMM) Laboratory, Canary Center at Stanford for Cancer Early Detection, Department of Radiology, School of Medicine, Stanford University, Palo Alto, CA 94304, USA; utkan@stanford.edu

**Keywords:** malignant ascites, epithelial ovarian cancer, chemoresistance, mechanical stress, flow-induced shear stress, photodynamic therapy, tumor microenvironment, transcoelomic metastases, tumor heterogeneity, epithelial to mesenchymal transition

## Abstract

**Simple Summary:**

Accumulation of excess fluid in the abdomen typically indicates abnormal function or disease, such as cancer, in the underlying tissues. This accumulation of fluid, or ascites, occurs more frequently in patients with advanced-stage ovarian cancer than any other type of cancer. The presence of ascites indicates the poorest outcomes for patients with advanced stage ovarian cancer, but little is known about the reasons for these dismal outcomes. This review discusses the current understanding of ascites, starting with an overview of ovarian cancer and ascites, followed by a description of the tools used to analyze the components of ascites and how these components modulate ovarian cancer biology. A perspective on the mechanical effects of ascites and the impact of mechanical stress on treatment resistance is provided. Lastly, treatment options for ascites and opportunities to develop new therapeutic strategies to improve outcomes are discussed.

**Abstract:**

Ascites refers to the abnormal accumulation of fluid in the peritoneum resulting from an underlying pathology, such as metastatic cancer. Among all cancers, advanced-stage epithelial ovarian cancer is most frequently associated with the production of malignant ascites and is the leading cause of death from gynecologic malignancies. Despite decades of evidence showing that the accumulation of peritoneal fluid portends the poorest outcomes for cancer patients, the role of malignant ascites in promoting metastasis and therapy resistance remains poorly understood. This review summarizes the current understanding of malignant ascites, with a focus on ovarian cancer. The first section provides an overview of heterogeneity in ovarian cancer and the pathophysiology of malignant ascites. Next, analytical methods used to characterize the cellular and acellular components of malignant ascites, as well the role of these components in modulating cell biology, are discussed. The review then provides a perspective on the pressures and forces that tumors are subjected to in the presence of malignant ascites and the impact of physical stress on therapy resistance. Treatment options for malignant ascites, including surgical, pharmacological and photochemical interventions are then discussed to highlight challenges and opportunities at the interface of drug discovery, device development and physical sciences in oncology.

## 1. Introduction

**Figure 1:**
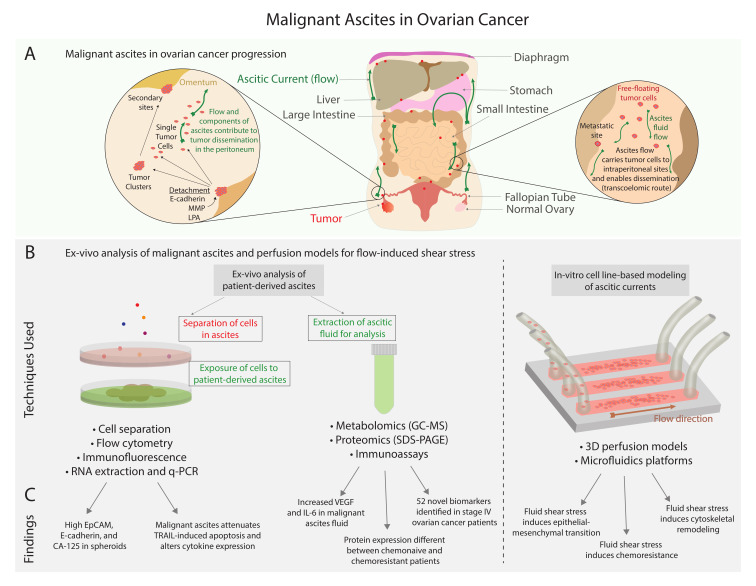
Malignant Ascites in the Dissemination and Progression of Ovarian Cancer. (**A**) Malignant ascites contributes to intraperitoneal tumor dissemination. Tumor cells detach from the primary tumor site as individual tumor cells or as tumor cell clusters, which can travel via currents of ascitic fluid to other intraperitoneal sites and create secondary cancer sites (transcoelomic route). (**B**) Ex vivo analysis of patient-derived ascites includes separation of cells using flow cytometry, RNA extraction, and metabolomic or proteomic analysis. In vitro cell line-based models of flow-induced shear stress reveal modulation of markers associated with aggressive disease. (**C**) Summary of key findings demonstrate increased pro-survival/attenuated anti-death pathways, enhanced metastatic potential and increased resistance to chemotherapy due to malignant ascites and flow-induced shear stress.

Compared to other gynecological malignancies (cervical, uterine, vaginal, and vulva), patients with ovarian cancer suffer from the highest mortality rates [1,2]. In 2021, an estimated 21,410 patients in the United States will be diagnosed with ovarian cancer and 13,770 will succumb to the disease [3]. By 2035, it is predicted that this number will rise by 67% to >250,000 patients [4,5]. Due to a lack of early detection methods, patients most often present with advanced-stage (III or IV) disease, limiting opportunities for therapeutic intervention. Advanced-stage ovarian cancer is frequently associated with the accumulation of fluid in the abdomen, known as ascites, which is comprised of cellular and acellular components. Cellular components of malignant ascites include tumor cells, which can exist as individual cells or spheroids, and stromal cells, which include fibroblasts, inflammatory cells, and endothelial cells [1,6]. The presence of these cell populations in ascitic fluid is indicative of underlying malignant tumors in the peritoneal cavity [1,7]. Acellular factors in malignant ascites include cytokines, such as interleukin (IL)-6 IL-8, proteins, and various metabolites [1,6]. These cellular and acellular factors provide tumor cells with a growth-promoting and immune-evading microenvironment, enabling phenotypic changes within tumor cell populations and facilitating chemoresistance. As shown in Figure 1, malignant ascites promotes tumor dissemination to intraperitoneal sites by means of biochemical and physical cues which have been modeled experimentally using ex vivo analysis of human samples and in vitro methods with cell lines [1,6,8,9,10].

**Figure 2:**
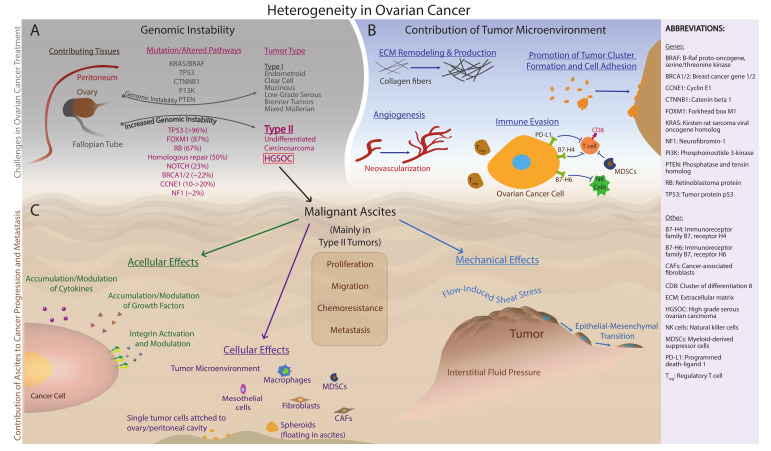
Illustration of Heterogeneity in Ovarian Cancer. (**A**) Contributing tissues and mutated or altered pathways in both type I and type II tumors are illustrated along with various subtypes included in each tumor type. Since HGSOC, a type II tumor, is the focus of this review, the frequency of mutated/altered pathways in type II tumors are noted as percentages, but the frequency of mutated/altered pathways in type I tumors has been omitted. (**B**) The tumor microenvironment also contributes to intertumoral heterogeneity via ECM remodeling through increased production of collagen, promotion of tumor cell cluster formation and adhesion to secondary intraperitoneal sites, increased angiogenesis through neovascularization, and immune evasion through the involvement of T_regs_, NK cells, and MDSCs. (**C**) The presence of malignant ascites is more frequently associated with type II tumors than type I tumors, and ascites contributes to a tumor-promoting microenvironment through acellular, cellular, and biophysical cues. Acellular factors, including cytokines, growth factors, and integrins, present in the ascites contribute to shaping the tumor microenvironment through enhanced tumor cell proliferation, dissemination, and colonization of distant sites. Cellular components of ascites, which include macrophages, fibroblasts, CAFs, mesothelial cells, MDSCs, individual tumor cells, and tumor cell spheroids, assist with immune evasion and ECM remodeling. Mechanical effects of ascites, notably flow-induced shear stress and interstitial fluid pressure, contribute to a tumor-promoting microenvironment through induction of EMT and increased expression of survival pathways that are indicative of aggressive and metastatic phenotypes, including chemoresistance.

In recent decades, research efforts have enabled an increased awareness of the critical role of ascites in ovarian cancer progression. While substantial progress has been made in the areas of cellular, acellular, and biophysical contributions, much of this work has yet to be translated to improvements in clinical outcomes. Since the most common primary type of cancer associated with malignant ascites is ovarian cancer [5,6,11,12,13,14,15,16,17,18,19,20,21,22,23,24], the focus of this review is the role of malignant ascites in ovarian cancer, specifically the contribution of ascites to advanced stage disease, modulation of the tumor microenvironment, remodeling of the extracellular matrix (ECM), and chemoresistance. In Section 2, major cellular and acellular components of ascites are introduced with a focus on the contribution of these factors to tumor growth, invasion, and metastasis. In Section 3, the impact of mechanical stress is examined from two different perspectives: (I) interstitial fluid pressure, and (II) flow-induced shear stress, particularly in 3D perfusion models for ovarian cancer. Finally, Section 4 describes the limited therapeutic options for the management of malignant ascites. Ovarian cancer is a collective term used to describe invasive cancers that originate from a variety of tissues that can include cells within and around the ovary, the fallopian tube, and epithelial cells that line peritoneal surfaces [25,26]. High-grade serous ovarian carcinoma (HGSOC) accounts for an estimated 70% of ovarian cancer cases, with endometrioid and clear cell cancers each accounting for 10% of cases, and mucinous tumors occurring at a rate of 3% [27,28]. Ovarian cancers are further divided into two subtypes: type I and type II (Figure 2A). Type I tumors often develop from the attachment of benign lesions to the ovary and include endometrioid, clear cell, low-grade serous, seromucinous, mucinous carcinomas and Brenner tumors [28,29]. In an updated report by Kurman and Shih [30], seromucinous tumors have instead been designated mixed Müllerian tumors, which include other benign or low-malignant potential ovarian tumors, in order to more appropriately represent morphologic and molecular features. Type II tumors are frequently associated with the presence of malignant ascites and include HGSOCs, carcinosarcomas, and undifferentiated carcinomas [25,29]. HGSOCs can be categorized based on morphology (the “usual type” or a solid, pseudoendometrioid, transitional variant) and gene expression (immunoreactive, differentiated, proliferative, or mesenchymal) [29,31]. Although the cell of origin in these tumors, particularly HGSOC, is unclear, both the ovarian surface epithelium (OSE) and secretory epithelial cells of the fallopian tube have been implicated [32,33]. Importantly, a major feature that distinguishes type I and type II tumors is the degree of genomic instability, defined as global deoxyribonucleic acid (DNA) copy number changes [25,29]. Type I tumors show limited chromosomal instability and often exhibit somatic mutations of phosphatidylinositol-4,5-bisphosphate 3-kinase catalytic subunit α (PIK3CA), catenin β1 (CTNNB1), phosphatase, and tensin homolog (PTEN), Kirsten rat sarcoma viral oncogene homolog (KRAS), B-Raf proto-oncogene, serine/threonine kinase (BRAF), mitogen-activated protein (MAP), extracellular signal-related kinase (ERK), and AT-rich interaction domain 1A (ARID1A) chromatin remodeling pathways [29,34]. Conversely, type II tumors display a higher degree of chromosomal instability compared to type I tumors, and most frequently harbor mutations in tumor protein 53 (TP53) along with alterations in homologous recombination repair, breast cancer gene 1/2 (BRCA1/2), retinoblastoma protein (RB), cyclin E1 (CCNE1), forkhead box M1 (FOXM1), and Notch3 pathways [29,35]. The most widespread subtype among type II tumors is HGSOC, which is responsible for 70%–80% of ovarian cancer-related deaths [29,31]. Since the focus of this review is primarily on type II tumors, notably HGSOCs, Figure 2A focuses on the frequency of mutated/altered pathways for type II tumors.

Metastases in ovarian cancer occur via three major routes of dissemination: transcoelomic (spread across the peritoneal cavity), lymphatic (spread through the lymph system), and hematogenous (spread through the blood) [1,36]. The most common route of dissemination is the transcoelomic route, which is responsible for the peritoneal metastases observed in about 70% of patients, likely due to the proximity of the ovaries to the peritoneal cavity [9,36]. Transcoelomic metastases are associated with increased mortality and morbidity in patients with ovarian cancer due to their ability to affect surrounding organs, such as those within the gastrointestinal tract [9,36]. Interestingly, transcoelomic metastases are also frequently associated with the production of malignant ascites [1,20,36]. It is unclear whether transcoelomic metastases are a passive process, in which tumor cells that possess metastatic characteristics are transported to other intraperitoneal sites via peritoneal fluid, or an adaptive process in which cells undergo resistance to anoikis, epithelial to mesenchymal transition (EMT), and alterations in integrin expression [36]. Spheroids, or aggregates of ovarian tumor cells, are also indicative of increased metastatic potential [16], since these cell clusters are able to evade host immunological defense mechanisms [36,37]. Even after the disaggregation of spheroids, tumor cells present in the ascites can evade immunological surveillance, and it has been suggested that malignant ascites may be the body’s response to intraperitoneal metastases due to the presence of immunomodulatory factors within ascitic fluid [36,38]. Additionally, the immunomodulators and growth factors residing in the ascites provide tumor cells with a favorable environment for growth and metastasis.

Transcoelomic metastases and malignant ascites commonly present in patients with advanced stage ovarian cancer, both of which are associated with increased mortality and morbidity [1,36]. Studies have also shown that the presence of ascites is associated with suboptimal cytoreduction as well as decreased progression-free and overall survival [5,39,40]. The current standard of care for treating ovarian cancer involves cytoreductive surgery in combination with platinum and taxane-based chemotherapy [41,42]. Initially, this regimen can lead to disease remission; however, the majority of advanced-stage tumors recur within the first two years [1,20]. Recurrence is often marked by clinical symptoms such as the presence of ascites and increased cancer antigen 125 (CA-125) levels or by computed tomography (CT) scans indicating tumor presence [5]. An alternative to this standard of care for patients with extensive disease consists of neoadjuvant chemotherapy (NACT) followed by interval debulking surgery [43,44]. Complete surgical resection provides patients with the best prognosis; however, this is not always possible, in which case debulking to achieve “optimal residual disease”, defined as the largest diameter of disease measuring ≤1.0 cm, is the next best option [45]. Altogether, the lack of early-stage diagnostics and effective therapeutics contribute to a dismal five-year survival rate of less than 30% for patients with advanced-stage ovarian cancer [20,46].

### 1.1. Heterogeneity in Ovarian Cancer

One major challenge in identifying effective targeted therapies for ovarian cancer is significant inter- and intratumoral heterogeneity. Intertumoral heterogeneity refers to patient-to-patient variation of ovarian cancer, making a standard of care treatment for all patients limited in effectiveness. Intratumoral heterogeneity in ovarian cancer includes differential cell morphology, gene expression, and metastatic potential, and will be the focus of this section [47,48]. Among the determinants of intratumoral heterogeneity in ovarian cancer are the inherent genomic instability as well as microenvironmental factors, as illustrated in Figure 2B. For example, using tissue microarray and k-means clustering analysis techniques, Tothill et al. [49] analyzed and classified tumor tissues from 285 patients with predominantly high-grade and advanced-stage serous or endometroid tumors of the ovary, fallopian tube, and peritoneum. The outcome of this investigation was the identification of four molecular subtypes of HGSOC (C1, C2, C4, and C5) each defined by unique molecular signatures. C1 tumors were associated with desmoplasia and had increased expression of stromal cell types [49]. C2 and C4 tumors were characterized by gene expression relating to immune cells, specifically demonstrating a high intra-tumor and intra-stroma presence of CD3+ lymphocytes [49]. C5 tumors showed overexpression of wingless-related integration site (Wnt)-related signaling genes, increased N-cadherin, increased P-cadherin, and decreased E-cadherin, indicative of EMT [49]. The results of this study suggest a role for unique tumor microenvironmental features associated with each HGSOC subtype to negatively impact both the extensive heterogeneity observed in ovarian cancer as well as patient outcomes [48,49].

### 1.2. Epithelial-Mesenchymal Transition in Ovarian Cancer

EMT refers to the phenotypic transition of a cell from an epithelial state to a mesenchymal state [9,50]. In ordinary ovarian processes, including postovulatory repair, cells in the OSE undergo EMT to aid in matrix remodeling [51,52]. Failure of these cells to switch to a mesenchymal phenotype can lead to the formation of inclusion cysts [51]. Consequentially, inclusion cysts can lead to the initiation of ovarian cancer [51,53]. Cancer cells undergoing EMT can lead to negative outcomes such as metastasis. Transition to a mesenchymal phenotype can be induced by microenvironmental cues [9,54,55] and this transition promotes cell motility, the ability to escape environmental stressors, [56,57] and the acquisition of resistance to traditional chemotherapeutic agents, including those used to treat ovarian cancer [58,59,60,61]. Metastatic cells that have undergone EMT can subsequently abandon their mesenchymal phenotype and undergo mesenchymal to epithelial transition (MET) upon colonization of a new site [51,54].

Interestingly, the presence of ascites can modulate the expression of various acellular factors, such as E-cadherin, contributing not only to EMT in ovarian cancer, but also the propensity of tumor cells to invade and metastasize [62]. A multitude of signaling factors such as epidermal growth factor (EGF) and SNAIL1/2 are thought to be involved in EMT [54,55]. In normal ovarian processes, such as postovulatory repair, EMT is induced by EGF, which enhances the activity of matrix metalloproteinases (MMPs), integrin-linked kinase, and ERK [51,53]. Additionally, SNAIL1/2 activation represses E-cadherin expression and has been shown to drive EMT in ovarian cancer, contributing to both disease relapse and lower survival rates [51,55]. Decreased E-cadherin levels are frequently observed in free-floating and metastatic ovarian cancer cells compared to primary tumors cells and is indicative of a more invasive phenotype [25,54,63]. As a result, downregulation of E-cadherin is a hallmark of EMT [64,65,66].

### 1.3. Prevalence and Symptoms of Ascites 

The presence of ascites is associated with several diseases including cirrhosis, heart failure, nephritis, pancreatitis, and cancer [7,67]. While cirrhosis accounts for 80% of ascites cases, cancer is the next leading cause (10%) [67]. In the context of cancer, malignant ascites is often indicative of peritoneal carcinomatosis, or the presence of malignant cells in the peritoneal cavity. Malignant ascites is most frequently observed in patients with advanced-stage ovarian cancer (nearly 40% of ovarian cancer patients with advanced disease), but also occurs in patients with pancreatic, colorectal, liver, and endometrial tumors [5,7,22]. Malignant ascites is also associated with extra-abdominal tumors, including those of the lung and breast; however, this is at a lower frequency [20].

A study by Ayantunde et al. [68] showed that symptoms of ascites in 122 patients included abdominal swelling (55%), abdominal pain (53%), nausea (37%), anorexia (36%), vomiting (25%), fatigue (17%), dyspnea (11%), early satiety (6%), and weight change (5%). Although ascites may be reduced by chemotherapy, intractable ascites, often found in patients with chemoresistant or recurrent disease, is associated with a poor prognosis [1,20]. Previous studies have shown that the frequency and volume of ascites increase with disease progression. With a total of 372 ovarian cancer patients, Ayhan et al. [69] found ascites present in 16.7% of patients with stage I or II disease compared to 46.3% of patients with stage III and IV disease. A study by Huang et al. [19] with 333 ovarian cancer patients cited incidence rates of 50.6% (stage I), 62.5% (stage II), 90.1% (stage III), and 100% (stage IV). This group also found the average volume of ascites to increase with stage of disease: 300 mL (stage I), 530 mL (stage II), 2460 mL (stage III), and 2810 mL (stage IV) [19]. Ascites volume also corresponded with patient survival; average survival of patients with <1800 mL ascites was 4.8 years while patients with >1800 mL of ascites was 2.4 years [19]. Similarly, Ayhan et al. [69] observed that median overall survival was 3.8 years without ascites, 2.3 years with <5000 mL of ascites, and 1.5 years in those with >5000 mL. The ascitic fluid volume also correlated with the number of tumor metastasis. Patients with < 3 regions of metastases had an average ascitic fluid volume of 700 mL while patients with > 3 sites of metastases had an average ascites volume of 3800 mL [19]. Interestingly, larger diameters of metastatic tumors were noted with increased volumes of ascitic fluid [19]. These findings collectively demonstrate that the presence of ascites in ovarian cancer is associated with advanced tumor stage, increased metastatic potential, and decreased survival.

### 1.4. Pathophysiology of Ascites and Its Role in Ovarian Cancer

The pathophysiology of ascites is multi-factorial. Proposed mechanisms include lymphatic obstruction and increased vascular permeability. Previously, it was hypothesized that the accumulation of peritoneal fluid is caused by intra-abdominal tumors obstructing lymphatic vessels [20,70]; however, studies in mouse models have demonstrated that ascites can form in the absence of lymphatic vessel obstruction [11]. Alternatively, growth factors (e.g., vascular endothelial growth factor (VEGF)) and cytokines (e.g., IL-6 and IL-8) present in the tumor microenvironment may increase vascular permeability and induce fluid accumulation [17,71,72,73]. Elevated vascular permeability can increase protein leakage, specifically that of serum albumin, which has an inverse relationship with ascitic volume [19,20]. Elevated levels of fluid production by the peritoneum have also been proposed as a mechanism for malignant ascites accumulation [15,74]. Additionally, there appears to be a correlation between the number of metastatic lymph nodes and the development of ascites [69].

In some cases, patients with disease that leads to ascites formation may present with bloody ascites. Bloody ascites, or hemorrhagic ascites, is defined as having a red blood cell count of > 10,000/μL in ascitic fluid and may occur spontaneously due to blood in the lymph [67,75]. Bloody ascites can also result from abdominal trauma or therapeutic procedures such as paracentesis [67,76]. Much of the literature on bloody ascites focuses on patients with cirrhosis or other liver diseases, such as portal hypertension; however, there are a few reports of bloody ascites in cancer patients, such as those with ovarian carcinoma [76]. The presence of bloody ascites in patients is considered a severe complication and is often associated with a poorer prognosis compared to patients with clear ascites [75,77].

In cirrhotic patients, bloody ascites (resultant from paracentesis) was significantly associated with higher kidney injury and mortality compared to cirrhotic patients without blood in the ascites fluid [75]. Massive bloody ascites resulting from ruptured intra-abdominal veins require aggressive therapeutic intervention while acute bloody ascites resulting from spontaneous bleeding occur more gradually and may not require treatment [76,77].

## 2. Malignant Ascites and the Tumor-Promoting Microenvironment

A variety of cellular and acellular factors present in the ascites of ovarian cancer patients contribute to tumor progression, metastasis, and immune evasion as depicted in Figure 2B,C. Cellular factors including tumor cells and stromal cells and acellular factors, such as cytokines and growth factors (summarized in Table 1), play key roles in regulating angiogenesis, immune and inflammatory responses, and proliferation.

### 2.1. Angiogenesis-Regulating Factors

Angiogenesis plays a key role in the normal functioning of many organs, including the ovary since follicular development is dependent on the formation of new vasculature [108]. Angiogenic activity is also a key characteristic of growing neoplasms and often begins once the mass reaches a certain volume (1–2 mm^3^) [108,109]. Since the demand for blood and oxygen increases as tumors increase in size, angiogenesis is a key factor in the progression of cancer [109]. Tumors can gain angiogenic capabilities through secreting various chemical signals, and these capabilities facilitate the growth and metastasis of the tumor [108,109]. In patients with cancers associated with ascites formation, tumors can often gain angiogenic properties through interacting with the cellular and acellular environment of malignant ascites. Within the ascites fluid and even plasma, notably in advanced-stage ovarian cancer patients, angiogenesis signaling proteins like VEGF and EGF are often present at elevated concentrations compared to patients with benign tumors or healthy controls [104,110,111]. Both signaling proteins have been shown to play key roles in the ability of ovarian tumors to grow and metastasize.

VEGF is a signaling protein essential for angiogenesis. In cancer, several studies have revealed a substantial role for VEGF mediated tumor growth, invasion, and metastasis [6,104]. In ovarian cancer, elevated VEGF levels are correlated with ascites development [105,106]. For example, Zhan et al. [104] found that VEGF levels were higher in malignant ascites (676.59 ± 303.86 pg/mL) compared to benign ascites (218.37 ± 98.15 pg/mL). High VEGF levels in malignant ascites were also associated with poor mean patient survival rates (8.3 ± 0.52 vs. 15.11 ± 0.66 months) [104]. Similarly, Santin et al. [110] showed that ascitic fluid from ovarian cancer patients contained high levels of VEGF. Santin et al. [110] also observed that plasma samples from ovarian cancer patients had higher VEGF levels than healthy patients and patients with higher grade malignancies had higher VEGF levels than patients with lower grade tumors. VEGF signaling can impose a myriad of tumor promoting effects including reduced Claudin-5 (tight junction protein) production [107] as well as increased microvessel density and MMP expression [106]. Altogether, these studies highlight that VEGF secretion by tumors plays a role in their growth, the production of ascites, and peritoneal dissemination [106,107,110].

Another angiogenesis-regulating factor is EGF. In general, the EGF receptor (EGFR) is a key molecular marker involved in cell proliferation, growth, and survival [80,81,82]. Dysregulation of EGFR-mediated signaling may lead to alterations in signaling pathways involved in cell cycle regulation, angiogenesis, and metastasis and is associated with a malignant phenotype for ovarian cancer [85,112]. In ovarian cancer, high EGFR expression is often associated with a more aggressive, invasive, and metastatic disease [83,84,85]. Lassus et al. [113] showed that increased copy number and overexpression of EGFR in serous ovarian carcinomas were associated with higher tumor grade, larger residual tumor size, and shorter overall and disease-free survival. Similarly, Psyrri et al. [82] found that in patients with ovarian cancer, EGFR expression levels are associated with decreased overall and disease-free survival. Other studies have shown that EGFR activation leads to a subsequent downregulation of E-cadherin [5,6,66,114], which is a hallmark for EMT and involved in metastatic cancer progression [54,115]. Overall, overexpression of EGFR in ovarian cancer patients is correlated with advanced-stage disease, high tumor grades, and increased metastatic potential.

The angiogenic capabilities of growing neoplasms can also be supported by the activation of the phosphoinositide 3-kinase (PI3K)-protein kinase B (AKT) pathway. The PI3K-AKT pathway is the most commonly activated pathway in cancers and plays an important role in reprogramming cellular metabolism [116]. In ovarian cancer patients, the PI3K-AKT pathway is frequently mutated or hyperactivated, enhancing the proliferative and adhesive properties of cancer cells [117]. Importantly, PI3K is activated by overexpression of receptor tyrosine kinases including VEGF and EGFR [117], both of which tend to be upregulated in patients with malignant ascites associated with advanced-stage ovarian cancer. Ascites have also been shown to induce angiogenesis through activation of the PI3K-AKT pathway in patients with liver cirrhosis [118]. This suggests the ability of ascitic fluid to contribute to disease progression by promoting angiogenesis and activation of pro-oncogenic pathways.

### 2.2. Adhesion-Regulating Factors

Integrins are cell adhesion receptors formed by specific combinations of two non-covalently bound type 1 transmembrane subunits, α and β. In humans, there are 18 α and 8 β subunits known, which can form 24 different heterodimer configurations [119]. Each subunit has a large extracellular domain that binds to ECM proteins, such as fibronectin, collagen, laminin, vitronectin, or counter-receptors of other cells. Intracellularly, the short cytoplasmic tail of integrins is linked to the actin cytoskeleton [120,121,122]. Integrins not only serve as adhesion molecules, but also enable cells to bidirectionally interact with their environment. In “outside-in” signaling, the ligation of an integrin leads to conformational changes to provide for the binding of signaling intermediates, such as enzymes and proteins. Integrin signaling is known to affect a variety of cellular behaviors including differentiation, growth and migration [120,123,124]. In cancer, aberrant integrin signaling is thought to influence behaviors including invasiveness, drug resistance and metastasis [125]. Targeting integrins is considered a promising treatment option, especially in the treatment of aggressive cancers, such as ovarian cancer [126].

The specific integrins proposed to be involved in ovarian cancer include α_2_β_1_ [127,128], α_4_β_1_, α_5_β_1_ [129], α_6_β_1_ [130], α_V_β_3_ [131,132], and α_V_β_5_ [131,132,133]. According to in vitro studies conducted on both primary cultures and ovarian carcinoma cell lines, the preferential adhesion of cells on type 1 collagen, which is abundant in the ovarian carcinoma microenvironment, is mediated by α_2_β_1_ integrin [127,128]. Scalici et al. [134] showed that, in xenograft models, tumor burden was significantly reduced when mice were treated with a combination of carboplatin and α_4_β_1_ integrin function-blocking antibodies or vascular cell adhesion molecule 1 (VCAM-1), a glycoprotein found on the surface of endothelial cells that is a ligand for integrin α_4_β_1_. Sawada et al. reported that EMT-associated downregulation of E-cadherin upregulates α_5_β_1_ expression, which mediates the attachment of ovarian cancer cells to the peritoneum and omentum [65]. The α_5_β_1_ integrin was also shown to be responsible for multicellular spheroid formation of OVCAR-5 cells in an in vitro model and adhesion of the same ovarian adenocarcinoma cell line to the peritoneum of athymic nude mice [135,136]. Further ex vivo studies by Hu et al. [129] revealed the correlation between α_5_β_1_ expression and drug resistance.

Malignant ascites can regulate integrin-mediated changes in ovarian cancer. Gillan et al. [131] studied the effect of periostin accumulation in malignant ascites during ovarian cancer progression. The results of the study suggested that periostin promotes invasiveness and migration of ovarian cancer cells in an α_V_β_3_ and α_V_β_5_-dependent manner. A follow-up study showed that blocking α_V_β_5_ integrins significantly reduced the activation of focal adhesion kinase (FAK) and Akt in the presence of malignant ascites, suggesting that Akt activation through the FAK pathway is α_V_β_5_-dependent [132]. Although Lane et al. [132] could not identify the ligand involved in Akt activation, they speculated that osteoprotegerin and periostin may be responsible for the ligation of α_V_β_5_.

Ahmed et al. [130] hypothesized that ascites-mediated alterations in integrin and urokinase plasminogen activator receptor (uPAR) expressions can trigger signaling pathways that regulate proliferation and invasion. A human OSE cell line (HOSE) and four ovarian cancer cell lines (HEY, PEO.36, OVCA433, and OVHS-1) were examined for their response to the presence of ascites. Among the integrin subunits that were evaluated, only α_6_ expression was significantly increased in response to ascites in all the cell lines, except HOSE. Expression of uPAR was significantly enhanced only in the invasive ovarian cancer cell lines OVCA433 and HEY, in contrast to the moderately invasive PEO.36, noninvasive OVHS-1, and normal HOSE cells, following stimulation by malignant ascites. The authors also assessed α_6_ and β_1_ integrin, and uPAR expression in patient tissues. β_1_ integrin was present in both normal and tumor tissue samples. uPAR expression was present only in tumor tissues. α_6_ integrin expression was significantly higher in high-grade tumor tissue samples than benign and low-grade tumors. The in vitro studies revealed that proliferation and adhesion of HOSE cells was not affected by ascites however, the presence of ascites significantly increased cell proliferation and adhesion in ovarian cancer cell lines: HEY, PEO.36, OVCA433, and OVHS-1. The proliferation of noninvasive OVHS-1 and invasive HEY cells was inhibited by antibodies against α_6_ and β_1_ integrins. Similarly, both antibodies could inhibit cell adhesion in all four cancer cell lines. The invasiveness of OVCA433 and HEY cells were enhanced by ascites, which was correlated with the increased cell surface urokinase plasminogen activator (uPA) and uPAR. In addition to antibodies against α_6_ and β_1_ integrins, invasiveness could also be inhibited by an antibody to uPAR in both OVCA433 and HEY cells. The MAPK/ERK (mitogen-activated protein kinase/extracellular signal-related kinase, MEK) pathway is a regulator of proliferation, adhesion, and invasion in cancer cells [130,137]. The study showed that ascites induces the activation of rat sarcoma (RAS) and downstream ERK pathway in all the cancer cell lines that were studied: HEY, PEO.36, OVCA433, and OVHS-1. The authors reported that the MEK pathway was blocked by antibodies to α_6_ and β_1_ integrins. Antibody against uPAR could also inhibit RAS activation in HEY, PEO.36, and OVCA433 cell lines but not in noninvasive cell line OVHS-1. These results suggest that ascites-mediated increase in α_6_β_1_ integrin and uPAR expression regulates cancer cell functions by activating oncogenic and survival pathways.

Another comprehensive study was conducted by Carduner et al. [133] to assess ascites-induced α_V_ integrin mediated increase in invasiveness and colony formation in ovarian cancer cell lines. First, the analysis revealed that ascites contained high concentrations of molecules involved in cell invasion and α_V_-mediated attachment: MMPs (2.74 ± 1.14 µg/mL), fibronectin (247.74 ± 6.65 µg/mL) and vitronectin (176.27 ± 21.57 µg/mL). The results suggested that the presence of ascites initiates deployment of α_V_ integrins to favor spheroid formation in IGROV-1 and migration in SK-OV-3 cells [133]. The results of the study showed that, in summary, integrin activation and deployment are favored in the presence of ascites, triggering cell signaling pathways important in cancer progression, invasion, metastasis, and immune disruption.

In addition to integrins, other factors present in the ascites can contribute to cell adhesion. A study by Uruski et al. showed that in the presence of malignant ascites, A2780 and NIH:OVCAR-3 cells adhered more to peritoneal mesothelial cells and peritoneal fibroblasts compared to cells exposed to benign ascites [24]. Additionally, it was determined that A2780 cell adherence was dependent on transforming growth factor (TGF)-β1 and hepatocyte growth factor (HGF) while that of NIH:OVCAR-3 cells was dependent on TGF-β1, growth-related oncogene-1 (GRO-1), and insulin-like growth factor-1 (IGF-1) [24]. Integrins were also examined in this study as well, and malignant ascites were found to upregulate α_5_β_1_ integrin expression on peritoneal fibroblasts [24]. These findings further support the role of malignant ascites in integrin-dependent and -independent ovarian cancer adhesion and progression.

### 2.3. Inflammatory and Immune Response Factors

Malignant ascites contains a milieu of pro-inflammatory factors that can contribute to ovarian cancer growth, metastasis, and the release of mucin 16 (MUC16), which is involved in ovarian cancer tumorigenesis and metastasis [78,86,93,138,139,140,141,142,143]. Pro-inflammatory cytokines and chemokines are a major component of ascites and modulate ovarian cancer in paracrine and autocrine fashions [144]. IL-6 and IL-8 are among the most abundant in ascitic fluid [145]. IL-6 plays a prominent role in innate and adaptive immune responses, and is thought to be involved in the transition from acute to chronic inflammation [146]. The inflammatory response from cancer cells promotes the infiltration of neutrophils, which benefits cancer progression via the secretion of IL-2, IL-6, IL-10, TNFα, and VEGF. The secretion of these factors leads to a higher neutrophil to lymphocyte ratio (NLR), which is correlated with decreased overall survival [147]. Reinartz derived a signal network model involving ovarian cancer cells and tumor associated macrophages (TAMs) linking IL-6, IL-10, and leukemia inhibitory factor (LIF) as cytokines that activate signal transducer and activator of transcription 3 (STAT3) [148]. IL-6, IL-10 and LIF were associated with early relapse, with IL-10 being the strongest indicator of poor outcome [148]. Additionally, IL-10, produced in substantial amounts by myeloid-derived suppressor cells (MDSCs), plays a role in creating a tumor-permissive tumor microenvironment [149,150]. Blockade of IL-10 signaling alleviates MDSC-mediated immunosuppression and improves survival, a unique role for IL-10 that is not redundant with other immunosuppressive molecules [149,150]. Blocking IL-10 activity also enhances cytotoxic T cell function in the peritoneal cavity and restricts tumor spread [151].

Bioactive lipids constitute a major class of soluble inflammation promoting mediators. Lysophosphatidic acid (LPA) is a bio-active phospholipid produced by autotaxin that activates six G-protein-coupled receptors [152,153]. LPA is a potential ovarian cancer biomarker that is present in 90% of stage I ovarian cancer patients and 100% of later stage patients [154,155,156]. Signaling through LPA receptors on ovarian cancer cells promotes migration and invasion [152]. Additionally, LPA promotes the production of prostaglandin (PG)E_2_, another bioactive lipid involved in ovarian cancer immunosuppression and progression [157].

Arachidonic acid (AA) and AA-derived metabolites are important components of ascitic fluid that can lead to ovarian cancer migration, invasion, and chemotherapeutic resistance, as well as immune suppression [158,159,160]. Transcriptomic analysis of the ovarian cancer ascitic microenvironment demonstrated that patients with high levels of phospholipase A2 group VII (PLA_2_G7), the enzyme that converts phospholipids to AA and leukotriene B4 (LTB4), were associated with a worse outcome as determined by regression-free survival. Additionally, there was a synergistic relationship between levels of AA and several cytokines including IL-6 and IL-10 such that patients with a high level of either IL-6 and AA, IL-10 and AA or TGF-β and AA had a worse outcome compared to those with a high concentration for only one mediator [148]. Functionally, AA derived from adipocytes, but not its metabolites PGD_2_, PGE_2_ or PGF_2α_, leads to the inhibition of cisplatin-induced apoptosis through the activation of Akt [161]. Metabolites of AA, including PGE_2_, PGF_2α_, TXB_2_ and LTB4, were identified in the ascites fluid of ovarian cancer patients over 30 years ago [162]. These metabolites, which include prostanoids, hydroxyeicosatetraenoic acids (HETEs) and leukotrienes, are produced via enzymatic cascades through either cyclooxygenases (COX) or lipoxygenases (LOX) [148]. In the COX pathway, AA is metabolized to PGH_2_ via COX-1 or COX-2 which is quickly converted by specific synthases to form PGE_2_, PGF_2α_, PGD_2_, PGI_2_, and TXA_2_ [163]. COX-1 and COX-2 have been identified as potential targets in ovarian cancer with the expression of each isozyme differing depending on the ovarian cancer subtype [164]. Ovarian epithelial cells with high COX-1 expression exhibit high levels of transcription factors: hypoxia-inducible factor 1-alpha (HIF-1α), VEGF, and VEGF receptor 2 (Flk-1) [165]. COX-2 has also been shown to play a role in ovarian cancer progression. COX-2 has been observed to be constitutively expressed in various ovarian cancer cell lines; introduction of PGE_2_ increases COX-2 expression, proliferation, invasion, reduction of apoptosis and secretion of EGF [166,167]. A major COX prostanoid, PGE_2_, signals through four different PGE_2_ receptors, EP1–4, with EP2 and EP4 linked to pro-malignant and immunosuppressive properties in ovarian cancer [168,169,170]. In primary ovarian cancer tissue, and multiple ovarian cancer cell lines, there is increased expression of EP4 and PGE_2_ exporter multidrug resistance protein 4 (MRP4) [171]. Interaction between platelets and tumors cells in the ovarian cancer tumor microenvironment leads to platelet enhanced ovarian tumor cell invasion, extravasation, and protection from host system defenses, while tumor cells serve as platelet agonists, increasing platelet adhesion, aggregation and degranulation [172].

The LOX pathway converts AA to HETEs or leukotrienes via multiple LOX enzymes [163]. Leukotrienes are primarily produced by stimulated leukocytes but can also be produced by epithelial and endothelial cells [163]. The 5-LOX enzyme leads to the creation of both 5-HETE and several leukotrienes that are involved in proliferation and inhibition of ovarian cancer apoptosis [173,174]. Expression of 5-LOX, 5-HETE and leukotrienes are correlated with the presence of TAMs in hypoxic areas of ovarian tumors [175,176,177]. Wen et al. [177] also demonstrated that 5-HETE and LTB4 promote macrophage migration, invasion, upregulation of MMP-7, and the release of TNF-α and heparin-binding epidermal growth factor-like growth factor (HB-EGF). LTB4 receptor 2 (BLT2) regulates ovarian cancer cell invasion and metastasis as well as cisplatin resistance [178,179].

Another bioactive lipid is sphingosine-1-phosphate (S1P) which has been identified in micromolar concentrations in ascites [180]. LPA and S1P stimulate the expression of IL-8 in ovarian cancer cell lines [181]. The sphingosine kinase/S1P receptor signaling pathway also plays a role in ovarian cancer migration, invasion and angiogenesis [182,183]. Inhibition of sphingosine kinase-2 with a selective inhibitor leads to decreased ovarian cancer cell survival, and induces apoptotic signaling and sensitization to paclitaxel treatment [184]. An additional inflammatory mediator is Cyr61 (cysteine-rich protein 61), a 40 kDa secreted matrix protein that stimulates proliferation, production of inflammatory mediator IL-6, prevents carboplatin-induced apoptosis and is associated with poor prognosis in ovarian cancer patients [185,186,187]. Significantly higher levels of Cyr61 were detected in malignant ovarian ascites, and in the serum of patients with ovarian serous cystadenocarcinoma [187].

#### 2.3.1. Innate Cellular Immune Response Factors

The immune system is increasingly recognized as a critical factor in ovarian cancer outcomes [188] and consists of both *innate immunity* (mediated, in part, by macrophages, NK leukocytes, complement, dendritic cells [DCs], MDSCs) and *adaptive* immunity (mediated, in part, by CD8+ cytotoxic T cells [T_c_], CD4+ helper T cells [T_h_], thymic and peripheral regulatory T cells [T_reg_], T helper 17 (Th17) effector cells, CD3 + CD56+ NK-like T cells, B cells) [189]. Ascites contribute to an immunosuppressive microenvironment, and the cellular immune populations they contain do not necessarily reflect those of the blood or tumor [190]. In one study, a higher proportion of CD8+ T_c_ (median 51.6%) was observed in tumor-infiltrating lymphocytes relative to peripheral blood leukocytes (23.9%), while ascites samples contained similar proportions of CD4+ T_h_ and CD8+ T_c_ (46.5% and 44.5%, respectively) [191]. Another study examining T cell diversity in the ascites of HGSOC patients compared to peripheral blood of post-menopausal healthy donors found that 3/15 patients had very low levels of CD3+ T cells, while the relative abundance of CD3+ T cells ranged from 25%–42% of total live CD45+ immune cells in 8/15 patients [192]. Additionally, flow cytometry data showed that HGSOC ascites contained a high proportion of activated CD4+ and effector memory T cells (CD4+ and CD8+) [192]. Further analysis examining dendritic cell presence and distribution found that DC presence increased in HGSOC ascites and that plasmacytoid DCs were the most represented subtype [192]. Additionally, when examining innate lymphoid cell (ILC) presence in HGSOC ascites, NK cells were found in higher proportion compared to ILC3s and lymphoid tissue inducer-like cells [192]. Specifically, Vazquez et al. [192] found a significant increase in the proportion of CD56^bright^ NK cells, which are compromised in cytolyzing cancer targets yet efficient at producing cytokines. Conversely, CD16+ NK cells, which are associated with cytotoxic responses, were significantly reduced in HGSOC ascites [192]. Thorough characterization of the ascites microenvironment as a distinct compartment is therefore imperative, as it is also believed to facilitate reversible dormancy within ovarian cancer cells that underlies recurrent disease [193] and may lend opportunities for improved therapeutic approaches.

##### Macrophages

Macrophages can be polarized to inflammatory *M_1_* (pathogen phagocytosis; tumor necrosis factor (TNF)-α, IL-1β, IL-6, IL-12, IL-23-associated, lipopolysaccharide+) or anti-inflammatory *M_2_* (wound healing and cellular proliferation; IL-4, IL-10, IL-13, arginase-associated, lipopolysaccharide-) phenotypes [194]. As chemotherapy induces tumor death, the subsequent release of lytic debris may then create an inflammatory environment that ironically promotes tumor growth mediated in part by macrophage release of bioactive lipids that stimulate COX pathways [195]. Reader et al. [196] showed that inhibition of EP4 receptors for the downstream product of COX enzymes, PGE_2_, can sensitize chemotherapy-resistant cancer cells that over-express class III β-tubulin to taxanes. EP4 is overexpressed in a variety of epithelial ovarian histologies [171] and upregulation of class III β-tubulin can be provoked by inflammatory stimuli [197] and is associated with aggressive biologic behavior in ovarian cancer [198,199]. Numerous other approaches to antagonism of the inflammatory milieu have been proposed [200]. Distinct macrophage populations in ovarian cancer ascites have been found to be prognostic of patient outcome. Ascites rich in CD163+ macrophages reduced recurrence-free survival [201]; CD163 is a scavenger receptor that internalizes hemoglobin–haptoglobin complexes but also interacts with erythroblasts and may be skewed towards an M_2_ phenotype. In a study using principle component and co-expression analyses of TAMs from ovarian cancer ascites, patients with TAMs that expressed an interferon signaling signature had a longer overall survival [202]. The presence of TAMs in the ovarian tumor microenvironment, however, has been associated with tumor progression and chemoresistance [203]. Studies have shown that TAMs secrete various soluble factors that induce invasive potential and chemoresistance [144,203,204,205,206]. TAMs can also interact with other immune components present in the tumor microenvironment to promote immunosuppression [203,207]. Additionally, TAMs have been shown to promote spheroid formation and tumor growth during early transcoelomic metastasis in an ovarian cancer mouse model [203,208]. In a study by Yin et al., [208] TAMs localized within spheroid centers and secreted EGF, causing an upregulation of integrins and VEGF signaling and supporting both tumor cell proliferation and migration.

##### Natural Killer (NK) Cells

NK cells mediate non-major histocompatibility complex (MHC)-restricted cytotoxicity and antibody-dependent cellular cytotoxicity (ADCC). In ascites, NK cells can be found in high density but often lack functionality [209]; among other mechanisms, IL-18 and TGF-β can decrease CD16 expression on NKs, impeding ADCC [210,211]. Diminished NK cell counts within ascites also correlate with chemoresistance [190]. NK cells may express estrogen receptors and programmed death protein (PD)-1 [211], suggesting additional mechanisms by which hormonal modulation and checkpoint inhibition may exert effects in gynecologic malignancies. Ex vivo expansion of NKs with autologous intraperitoneal delivery is a promising, nascent therapeutic approach [212].

##### Dendritic Cells (DCs)

DCs are among the most potent antigen-presenting cells. Immature DCs phagocytose antigens readily but require maturation by cytokine stimulation to become maximally stimulatory towards T cells [213]. DCs prime naïve T cells to activate antigen-specific immunity and consist of *myeloid-derived* (DC_1_; interferon-gamma [IFN-γ] and IL-12-associated; blood dendritic cell antigen (BDCA)-1^+^ BDCA-3^+^ CD16^+^) and *plasmacytoid-derived* (DC_2_; TNF-α and IL-8-associated; capable of inducing neovascularization; CD123^+^ BDCA-2^+^) lineages. Ovarian cancer ascites is enriched with DC_2_ but void of DC_1_ [214]. Interestingly, the number of DCs in ascites of chemo-naive high-grade serous ovarian cancer patients does not correlate with survival [215], suggesting deficits in functionality may be of greater importance. Notably, DCs express the PGE_2_ receptors EP2 and EP4, and PGE_2_ compromises toll-like receptor-mediated DC activation [216], suggesting one mechanism by which the inflammatory environment of ascites may lead to DC malfunction.

##### Myeloid-Derived Suppressor Cells (MDSCs)

Immunosuppressive MDSCs represent a minor population in healthy individuals, but emerge in larger numbers during inflammatory conditions [217]. Cells of this lineage exert their immune suppression through arginase, inducible nitric oxide synthase (iNOS), TGFβ, IL-10, COX2, indoleamine 2,3-dioxygenase (IDO), sequestration of cysteine, and a decrease in L-selectin expression by T-cells [217]. Studies have shown that ascites in tumor-bearing mice have significantly higher amounts of MDSCs compared to naïve mice, and that the presence of MDSCs in ascites increases with disease stage [218]. Inhibition of MDSCs improved survival in a syngeneic mouse model of ovarian cancer using ID8-fLuc [218].

#### 2.3.2. Adaptive Immune Response Factors

##### T Helper (T_h_), Cytotoxic T (T_c_), and T Helper 17 (T_h_17) Cells 

T_h_ cells are MHC class II-restricted and can be separated into two classes depending upon specific cytokines the cells secrete in response to antigenic stimulation [219]: (1) *T_h_1*, associated with a proinflammatory response important for killing intracellular parasites and perpetuating autoimmune response via IL-2, IFN-γ, TNF, granulocyte-monocyte colony stimulating factor (GM-CSF), and IL-3 in response to antigen-presenting cells, and (2) *T_h_2*, linked to an anti-inflammatory response, immunoglobulin (Ig) E and eosinophilic responses via IL-4,-5,-13, and -10 [220]. Th_1_ shifts enhance apoptosis of ovarian cancer cells [221], and the ratio of TNF-α to IL-4 correlates with patient survival [222].

T_c_ cells are MHC class I-restricted. Over-expression of PD-L1 (programmed death ligand-1), the target of drugs such as atezolizumab, durvalumab, and avelumab, in malignant ascites is associated with T_c_ malfunction and blunting of the immune response [223]. Expression of PD-1, the target of agents, such as nivolumab and pembrolizumab, is a hallmark for T cell exhaustion, at which point T cells sub-optimally secrete cytokines and lose cytotoxicity, and are eventually eliminated from the tumor microenvironment [224]. In one study, expression of programmed cell death protein 1 (PD-1) was 30% in ascites-derived T cells and 50% among non-immune cells [215]. Immuno-oncology as a strategy to exploit the immune system remains an active area of interest for ovarian tumors [225]. CD4 + T_c_ and CD8 + T_h_ cells isolated from ovarian cancer ascites demonstrate increased X-box binding protein 1 (XBP1) messenger ribonucleic acid (mRNA) splicing compared with peripheral T cells from cancer-free patients [226], reflecting endoplasmic reticulum stress and activation of the unfolded protein response (UPR) that controls mitochondrial respiration and anti-tumor function. Increased CD4+ to CD8+ population shifts [145] and decreased IL-17 from Th17 effector T cells [227] within ovarian cancer ascites have been associated with compromised survival. Th17 effector cells secrete chemokine (C-X-C motif) ligand (CXCL)9 and CXCL10 to recruit tumor-infiltrating lymphocytes. Th17 cells decrease with advancing stage, represent only a minority of T cell subsets, and appear to be inversely related to T_regs_ [227].

##### Regulatory T Cells (T_regs_)

CD4+CD25+ T_regs_ blunt the immune response via co-stimulatory molecules such as B7-H4. Thymic T_regs_ express FOXP3 (forkhead box protein 3), and peripheral T_regs_ express FOXP3 only upon induction [228]. T_regs_ display a higher degree of activation in the ascites than in blood, and the number of T_regs_ correlates with the proportion of epithelial cell adhesion molecule (EpCAM)+ cancer-derived epithelial cells in ascites fluid [229]. Additionally, T_regs_ increase with stage [230]. In an ascitogenic mouse model [231], treatment with a low-dose anti-CD25 antibody improves survival and results in lower intraperitoneal tumor volumes when mice are challenged with WF-3 cells [230]. T_regs_ isolated from ascites compared to peripheral blood demonstrate increased suppressive capacity marked by a distinct cell surface expression profile, which includes high levels of CD39, CD73, TGF-β and GARP (glycoprotein A repetitions predominant) [232]. The inflammatory mediator PGE_2_ has been implicated in conversion of T_h_ to T_reg_ dominance [233]. Hypoxia within intraperitoneal tumors can promote tolerance through recruitment of T_regs_ via CCL28 [234]. IL-6 present in ascites promotes TNF receptor 2 expression and stimulates the immunosuppressive capacity of T_regs_ [235]. Depletion of T_regs_ thus has the potential to be a powerful immunotherapeutic approach to malignant ascites in ovarian cancer.

### 2.4. Lysosomes, Secreted Vesicles, and Ascites

Lysosomes play a key role in regulating cellular homeostasis and recent studies have implicated alterations in lysosomal signaling in a variety of diseases including cancer [236]. As a mediator of cell catabolism, lysosomes are critical for nutrient sensing and frequently associate with the rapamycin complex 1 (mTORC1) [236,237]. Since lysosomes play a role in nutrient sensing, it is thought that lysosomal signaling may help cancer cells meet increased energy demand [236]. Indeed, studies have shown that the tumor cells rely on lysosome degradation and recycling processes for nutrients, in a variety of cancers [236,238]. Lysosome signaling may also contribute to cancer progression through enhanced proliferation and metastatic capabilities [236].

Ascites may contribute to lysosomal signaling dysregulation since studies have shown that ascitic fluid can contain high expression levels of lysosomal enzymes and genes. In a study examining the activity of lysosomal enzymes in the ascites of patients with gynecologic cancers and pelvic inflammatory disease, it was found that β-glucuronidase, β-galactosidase, and α-mannosidase levels were increased in patients with ascites related to malignant disease compared to those with no disease or benign ovarian cyst fluid [239]. Other studies have explored the association between expression levels of lysosome-associated membrane protein-1 (LAMP1), which protects the lysosomal membrane from intracellular proteolysis [240], and ovarian cancer progression. Meunier et al. found that in the presence of ascites, a tumorigenic ovarian cancer cell line had upregulated expression of LAMP1, and LAMP1 expression significantly correlated with the effect of ascites on cell migration [241]. Another study examining LAMP1 expression in ovarian cancer patients found that LAMP1 expression was significantly higher in tumor tissues compared to benign or normal tissues [240].

In the context of ovarian cancer, lysosomal signaling has also been implicated in aggressiveness and chemoresistance. In a study by Fang et al. [242] exploring the mechanism behind ovarian cancer stemness and chemoresistance, it was shown that downregulation of tyrosine kinase phosphorylation increased Notch3 degradation via a lysosomal pathway, therefore implicating the lysosome in ovarian cancer tumorigenicity. Lysosomes also play an important role in the secretion of exosomes, and certain regulators of exosome secretion are associated with cancer progression and metastasis [243,244]. In a study by Dorayappan et al. [244], altered lysosomal phenotypes were observed under hypoxic conditions in various ovarian cancer cell lines and patient-derived ascites. Altered lysosomal phenotypes in these cells not only increased exosome release but also decreased endolysosomal fusion [244]. This observation is significant because cisplatin, the standard of care for ovarian cancer, concentrates in lysosomes. Alterations in lysosomal pathways have been implicated in decreased cisplatin cellular uptake and cisplatin-resistance [244,245].

The role of tumor-derived extracellular vesicle (EV) secretion in ovarian cancer progression and response to therapy, was examined by Alharbi et al. [246]. It was observed that low oxygen tension led to an increase in the expression of hypoxia-related proteins and induced EV release. Importantly, the authors noted that EV secretion differed across ovarian cancer cell lines. Hypoxia-related proteins that were examined were mainly involved in metabolic reprogramming, specifically relating to the glycolytic pathway. Authors also reported that platinum-resistance significantly increased in normoxic cells when exposed to EVs secreted by hypoxic cells [246]. When tumor-derived EVs were isolated from patients with ovarian cancer, Alharbi et al. found similar results in that they contained glycolytic pathway-related proteins and were most enriched in patients with recurrent disease [246]. These findings suggest that EVs containing glycolytic pathway-related proteins can transmit chemoresistance to other tumor cells, facilitating the progression of disease [246].

### 2.5. Proliferation Regulating Factors

A fundamental characteristic of cancer cells is the propensity for sustained, chronic proliferation. While growth-promoting signals are tightly regulated by healthy tissues to control cell division and maintain tissue architecture, cancer cells deregulate these signals, enabling unchecked proliferation [56]. Ovarian cancer ascites is a repository for tumorigenic factors, facilitating deregulation and providing a microenvironment that is conducive to aberrant tumor growth. Soluble factors in ascites, including cytokines, proteins, metabolites, and exosomes, mediate autocrine and paracrine signaling among tumor cells and stromal cells [6]. In addition to these acellular factors, ascites is also a reservoir for tumorigenic cellular components, including cancer cells and cancer-associated cells. Lactate dehydrogenase (LDH), is an enzyme that is overexpressed in malignant ascites and plays a key role in aerobic glycolysis through the bidirectional conversion of pyruvate to lactate [247,248]. Cancer cells often utilize aerobic glycolysis (also known as the Warburg effect), instead of mitochondrial respiration, to generate adenosine triphosphate (ATP) and fuel tumor growth [249,250]. In addition to LDH, a variety of factors that support cancer cell metabolic activity and proliferation accumulate in ascites. Taken together, these results illustrate that metabolism-altering, pro-proliferative factors in ovarian cancer ascites contribute to disease recurrence.

EVs comprise another class of factors that are abundant in malignant ascites and can contribute to cancer cell proliferation. EVs are important mediators of crosstalk between cancer cells and their microenvironment, and are thought to be involved in peritoneal metastasis [251]. Ovarian cancer ascites contain distinct populations of exosomes that carry unique cargo, including proteins and microRNAs (miRNAs), that promote cancer cell proliferation, invasion, and angiogenesis [252]. Ascites-derived exosomes have also been shown to have largely immunosuppressive effects that enable cancer cells to bypass the host response. In a study by Taylor et al., [253] the effects of ovarian cancer ascites-derived exosomes on Jurkat cells (T lymphocytes) were investigated. Jurkat cells incubated with exosomes had diminished expression of CD3-*ζ* and Janus Kinase 3 (JAK 3), which are involved in T-cell activation, and underwent increased DNA fragmentation. Exosomes have also been shown to mediate the transformation of host cells into tumor-promoting cells, such as cancer-associated fibroblasts (CAFs) and TAMs [252]. Wei et al. [254] showed that malignant ascites-derived exosomes increased the expression of CAF-specific markers, including fibroblast activation protein (FAP), alpha-smooth muscle actin (α-SMA), and fibronectin, in peritoneal mesothelial cells (MCs). Ultimately, these transformations facilitate the construction of a microenvironment that is conducive to cancer cell proliferation and the protection of cancer cells from immune response.

In addition to numerous acellular factors, various cellular components in ascites orchestrate cancer initiation and progression. These cellular components include cancer cells, along with tumor-associated stromal cells. In a study by Latifi et al., [62] ascites-derived cells from chemonaïve and chemoresistant patients were stratified into adherent and non-adherent populations, then characterized and compared. Adherent cells had enhanced proliferative capacity, significantly higher vimentin and MMP9 mRNA levels, and shared an antigen profile with stromal fibroblasts and mesenchymal stem cells. Relative to adherent cells, non-adherent cells expressed significantly higher mRNA levels for E-Cadherin, EpCAM, STAT3, and octamer-binding transcription factor 4 (Oct4), which are markers for disease progression. For example, STAT3 modulates ovarian cancer cell proliferation by regulating the expression of genes such as cellular myelocytomatosis (c-Myc), cyclin D1, B-cell lymphoma-extra-large (Bcl-xL), and surviving [255]. Oct4 is a transcription factor that regulates the self-renewal of pluripotent stem cells, is enriched in tumor-initiating cells, and is also a key promoter of ovarian cancer cell growth [256,257,258]. A study by Ruan et al. [259] showed that in a subpopulation of Oct4-overexpressing ovarian cancer cells, knockdown of Oct4 caused a significant decrease in proliferation, and an increase in sensitivity to cisplatin. Conversely, inducing overexpression of Oct4 in cell populations with lower endogenous Oct4 led to significantly higher proliferation, and a decrease in sensitivity to cisplatin. Overall, these results provide important insights into the phenotypic and tumorigenic heterogeneity of cell populations that are present in ascites and contribute to disease progression.

A variety of cancer-associated cells in malignant ascites, particularly TAMs and CAFs, participate in complex intercellular communication and potentiate disease progression. TAMs can comprise more than 50% of the cell population in malignant ascites from ovarian cancer [260]. TAMs play a central role in disease progression through the expression of molecules that promote angiogenesis, suppress the immune system, facilitate invasion, and induce cancer cell proliferation [261,262]. The polarization of circulating monocytes into TAMs occurs in ascites due to the presence of various soluble factors, including chemokine ligand (CCL)2 and colony stimulating factor-1 (CSF-1). Ascites-derived TAMs closely resemble immunosuppressive, M2-like macrophages, through their expression of M2 markers, such as CD163, CD204, CD206, and IL-10 [207]. Tumor cells and TAMs coordinate through bi-directional signaling to create an immunosuppressive, tumorigenic microenvironment. A study by Takaishi et al. [263] demonstrated that THP-1 cells (human monocytic cells) were polarized to an M2 phenotype via STAT3 activation when cultured with ovarian cancer ascites. In a study by Hagemann et al., [264] ovarian cancer cells were co-cultured with macrophages to elucidate changes in gene expression as a result of heterocellular crosstalk. Macrophages co-cultured with cancer cells showed significant upregulation (50- to 500-fold increase) of a variety of genes, such as CCL2, TNF- α, VEGF-C, and CSF-1, among many others. Co-cultured macrophages also showed increased expression of scavenger receptors and mannose receptors, which are both markers for alternatively-activated, or M2, macrophages.

CAFs are another class of cancer-associated cells that comprise ascites and contribute heavily to the initiation and progression of ovarian cancer. Quiescent fibroblasts can become activated as part of the host response to injury [265]. However, cancer cells recruit and reprogram resident fibroblasts into CAFs through the production of various factors, such as TGF-β and PDGF [266,267,268,269]. Additionally, cancer cells can dysregulate miRNA expression in fibroblasts, initiating their transformation into CAFs [270,271]. Tumor-secreted microvesicles can contain miRNAs that function as signaling molecules, influencing target cell phenotype and modulating the tumor microenvironment and metastatic niche [272]. While cancer cell-secreted factors strongly influence fibroblast phenotype, the communication is bidirectional; CAFs can, in turn, modulate cancer cell phenotype and the tumor microenvironment. Specifically, CAFs functionally contribute to tumor progression through a variety of mechanisms, including ECM remodeling, induction of EMT, promotion of chemoresistance and modulation of cancer stem cell populations, among many others [268]. The effects of CAFs on ovarian cancer proliferation and invasion are, in part, mediated through the release of growth and ECM remodeling factors. In a study by Cai et al., [267] Ovarian cancer-activated fibroblasts overexpressed hepatocyte growth factor and MMP-2, inducing functional changes in ovarian cancer phenotype, such as increased adhesion and invasion. Additionally, in a recent study by Gao et al., [273] HGSOC-derived ascitic spheroids were shown to possess a distinct architecture, where EpCAM^+^ epithelial cells surrounded a core of CAFs. The adhesion between ascites tumor cells and CAFs was mediated by integrin α5, and CAF-released EGF promoted and maintained integrin α5 expression on tumor cells, further potentiating spheroid formation. Importantly, spheroids composed of SK-OV-3 cells and CAFs (heterospheroids) displayed the strongest adhesive capacity and lowest apoptosis rate compared to SK-OV-3-only spheroids and single cells. These results highlight the critical role that CAFs play in ovarian cancer proliferation and metastasis.

### 2.6. Metabolomic and Proteomic Profiling of Ascites Fluid and Blood in Ovarian Cancer

Genomics, transcriptomics, proteomics, and metabolomics have dramatically enhanced our ability to unravel communication networks by examining changes in gene, transcript, protein and metabolite expression, respectively. These emerging “omic” techniques bring to light molecular interactions associated with pathology, and can guide the development of precision medicine [274,275,276]. Commonly, sources of biomarkers include blood, urine, stool, lymph, and tissue [277,278]. In ovarian cancer, omics methodologies have been applied to cell lines [279], blood [280], exosomes [281], tissues [282], and ascitic fluid [283,284,285].

There are several benefits of probing ascites for biomarkers in terms of accessibility and knowledge which can be acquired. First, ascitic fluid is frequently drained via paracentesis, catheter, or shunt; therefore, samples can be obtained in accordance with regular procedures [286]. Second, the onset of ascites is frequently related to poor prognosis [6,20]. By analyzing soluble factors in ascitic fluid, modulations in cell behaviors such as an EMT or chemoresistance can be identified [1,6]. Third, in vivo, ascitic fluid imparts fluid shear stress on tumors; thus, one can deduce cell phenotypic effects due to mechanical stress [287,288].

As summarized in Table 2**,** several published studies substantiate changes to the metabolomic and proteomic profiles of malignant ascites. For instance, Shender et al. [285] revealed 41 metabolites and 424 proteins unique to malignant ascites, compared to non-malignant cirrhosis ascites. Kuk et al. [284] sampled ascites fluid from stage IV serous ovarian carcinoma patients. By distinguishing ascites fluid proteins from serum proteins, the authors confirmed expression of 25 protein biomarkers associated with ovarian cancer, along with 52 novel proteins [284]. Recently, Ahmed et al., [283] utilized a label-free quantitative proteomic approach to characterize ascites from chemonaïve and chemoresistant patients. Compared to ascites from patients with chemonaïve tumors, expression of 178 proteins was diminished and 175 proteins was enriched in ascites from patients with chemoresistant tumors [283]. Several of the proteins that were comparatively overexpressed in ascites from patients with chemoresistant tumors, such as acetyl-CoA carboxylase (ACC), glycine dehydrogenase (GDLC), and squalene synthase (FDT1), play pivotal roles in cellular metabolism. GLDC, for example, induces dramatic changes in glycolysis and glycine/serine metabolism, drives tumor-initiating cells (TICs), and promotes tumorigenesis in non-small cell lung cancer [283,289]. Additionally, ACC drives de novo fatty acid biosynthesis, enabling rapid construction of cell membranes to sustain swift cancer cell proliferation rates [290]. Dysregulated fatty acid biosynthesis has also been shown to promote cancer stemness in multiple cancers, including ovarian cancer [291,292]. Similarly, FDT1 is a key enzyme in cholesterol biosynthesis, which plays an important role in cancer stem cells and tumorigenesis [293].

Mapping protein and metabolite behavior can facilitate an in-depth understanding of signaling transduction networks altered in disease progression. Modulations in Wnt [294], TGF-β [295], TP53 [296], MAPK [297], and PI3K/PTEN/AKT [117] are implicated in ovarian cancer progression and chemoresistance. Success in pinpointing molecular targets within signaling networks has the potential to identify novel therapeutics and combat disease progression.

**Table 2:** Proteomic/Metabolomic Profiling on Ovarian Cancer Ascites.

Reference	Major Findings
Kuk et al., 2009. [284]	Utilized mass spectrometry to analyze proteins from ascites fluid of stage IV serous ovarian carcinoma patients Confirmed 25 biomarkers associated with ovarian cancer and identified 52 novel potential biomarkers
Elschenbroich et al., 2011. [298]	Analyzed ascitic fluid from serous ovarian cancer patients using comprehensive shotgun proteomics 51 candidate proteins identified using mRNA transcriptomic integration Quantitative proteomics on candidate proteins by systematic selected reaction monitoring-mass spectrometry (SRM-MS) and stable isotope dilution-SRM (SID-SRM)
Shender et al., 2014. [285]	Compared malignant ascites and cirrhosis ascites 41 metabolites unique to malignant ascites identified using gas chromatography–mass spectrometry 424 proteins specific for malignant ascites found using sodium dodecyl sulfate-polyacrylamide gel electrophoresis (SDS-PAGE)
Ahmed et al., 2016. [283]	Label-free quantitative proteomic approach to identify ascites differences before chemotherapy treatment and after treatment/at disease recurrence Compared to chemonaïve tumors, 178 proteins were diminished in chemoresistant tumors 175 proteins were enriched in expression in chemoresistant tumors

The ascitic proteome provides important insights into potential biomarkers to detect ovarian cancer. Many factors found in ascites are also found in the serum or plasma of ovarian cancer patients. Kuk et al. [284] analyzed the ascitic and plasma proteomes in ovarian cancer patients and found that ~35% of proteins identified in ascites were also found in the plasma. While this does not mean that the remainder of the observed proteins are unique to ascites, it does suggest that the composition of ascites and plasma in ovarian cancer patients differs [284].

CA-125 levels are typically elevated in the ascites and plasma of ovarian cancer patients. Monitoring plasma CA-125 levels may also provide prognostic information about disease state. In one study, a 50% decrease in CA-125 levels was observed in patients with chemosensitive disease (relative to levels prior to the initiation of treatment), in contrast to patients with disease progression or chemoresistance in whom CA-125 levels were elevated following treatment [299]. A continuous rise in CA-125 levels is typically indicative of disease recurrence [299]. A study examining CA-125 levels in platinum-sensitive and platinum-resistant ovarian cancer patients corroborated these general trends in CA-125 levels after treatment [300]. In platinum-sensitive patients, CA-125 levels decreased from 607.37 ± 183.13 U/mL to 21.87 ± 6.59 U/mL after treatment [300]. CA-125 levels in platinum-resistant patients increased from 915.8 ± 373.87 U/mL before treatment to 4211.95 ± 2105.98 U/mL after treatment [300].

Additional cytokines and signaling proteins such as ILs may also be present in the ascites and plasma of ovarian cancer patients. One study showed that in pre-operative ovarian cancer patients, serum IL-6 levels were higher compared to controls or patients with a benign tumor [301]. Serum IL-8 and IL-10 expression levels have also been reported to be higher in patients with both benign and malignant ovarian cancer compared to controls, and elevated prior to chemotherapy compared to after chemotherapy [302]. The serum levels of other previously reported ascitic components, such as EGF, LPA, and uPA have also been reported to be elevated in ovarian cancer patients compared to controls [111,303,304]. Pre-operative levels of mesothelin, a mesothelial cell surface protein, were also reported to be elevated in ascitic fluid and plasma from ovarian cancer patients compared to patients with benign ovarian tumors or controls [305]. These findings suggest that proteomic profiling of both ascitic fluid and plasma of ovarian cancer patients may help identify potential therapeutic options to combat disease progression.

### 2.7. Magnitude and Directionality of Ascites

Biophysical forces, such as flow-induced shear stress from currents of ascitic fluid, can confer mechanical effects on tumors and the surrounding microenvironment. Ascites can also carry tumor cells, aggregates, signaling molecules, and EVs to distant intraperitoneal sites through convection, thereby promoting tumor invasion and metastasis. Characterizing the flow pattern of ascites is important to understanding the tumor microenvironment and distribution of intraperitoneal metastasis and may help in creating more effective targeted therapies in the treatment of malignant ascites or metastatic ovarian cancer.

Historical perspectives on the flow pattern of ascites in the peritoneal cavity suggest the role of various watersheds within the peritoneum that drive directional fluid flow with the help of gravity and subdiaphragmatic pressure [306,307]. Meyers [306] examined the role of ascites flow in intraperitoneal malignant seeding and concluded that the flow of ascites occurs along specific pathways in the abdomen based on the contribution of factors such as gravity and subdiaphragmatic pressure. When examining peritoneal fluid flow of 35 supine patients, Meyers found that fluid in the inframesocolic compartment, left infracolic space, and right infracolic space preferentially flow to specific areas of the lower abdomen near the small bowel or pelvis [306]. Fluid then ascends the paracolic gutters and ultimately reaches the right subhepatic and subphrenic spaces [306]. Based on this work, four sites of metastases were identified based on ascitic fluid flow and the effect of flow, gravity, and variation of intraperitoneal fluid pressures on intraperitoneal seeding were identified [306]. Another study examining intraperitoneal fluid movement throughout the peritoneal cavity found that three main abdominal barriers (longitudinal, upper transverse, and lower transverse) act as watersheds and assist in the spread of intraperitoneal effusions [307].

Rather than biologic features, such as tumor aggressiveness, driving this widespread distribution, physical factors, notably intraperitoneal fluid hydrodynamics and gravity, were implicated [308]. Specifically, studies have shown that intraperitoneal hydrodynamics direct fluid to subdiaphragmatic spaces, which may also be responsible for the invasion of upper abdominal spaces by cancer cells suspended in the ascites [308,309]. This is also supported by the fact that cells are often limited to more local implantation near the primary site of cancer when ascites is not present [308,309,310].

Other studies have utilized the findings of Meyers to understand the effects of peritoneal fluid flow on endometriotic lesions, which frequently vary by distribution pattern and type of lesion (peritoneal endometriosis, ovarian endometriomas, and deeply infiltrating endometriosis) [311]. Bricou et al. [311] concluded that one main reason for the observed asymmetric distribution of endometriotic lesions is the inherent anatomical asymmetry in the pelvis. Additionally, Bricou et al. [311] also suggested that, since lesions commonly occur in the Pouch of Douglas, that gravity may also be playing a crucial role in the distribution of endometrial lesions.

More recent studies on intraperitoneal dissemination of cancer and the role of peritoneal fluid flow in this process have concluded that more frequent tumor dissemination occurs in organs with peritoneal fluid resorption, which include the omentum and omental appendages [308,310]. On the other hand, organs such as the small bowel showed reduced tumor number, likely due to peristaltic motion [308]. Similar to previous findings that intraperitoneal fluid contributes to the dissemination of ovarian tumor cells, Carmignani et al. [308] showed that in patients with mucinous adenocarcinoma, increased distribution of cancer cells throughout the abdomino-pelvic cavity was frequently observed in the presence of mucus ascites.

One important limitation to note is that in many of the studies on intraperitoneal fluid flow pattern, the patients tended to be supine and/or anesthetized. While these studies provided a groundwork for patterns of peritoneal fluid flow, it is unclear how similar those findings would be to the behavior of ascites flow in patients who are upright and mobile. In fact, it has been noted that pressure in the lower abdomen is almost three times higher when a patient is in the upright position compared to the supine position [306,312]. For this reason, further research into the flow pattern of ascites in patients is needed for the development of effective targeted therapies for the treatment of malignant ascites and metastatic ovarian cancer.

## 3. Interstitial Fluid Pressure and Fluid Stress

Peritoneal surfaces, including those of the ovaries and fallopian tubes, are regularly exposed to shear forces and fluid pressures due, in part, to organ mobility, negative sub-diaphragmatic pressure and bowel peristalsis [9,10,287,313]. Changes in shear forces and fluid pressures in the peritoneum due to the accumulation of ascites remain to be quantified and characterized; however, the presence of ascites has been shown to negatively impact the prognosis for patients with ovarian cancer [16,287,313]. Increased interstitial fluid flow (IF) and interstitial fluid pressure (IFP) may also contribute to tumor progression and to the modulation of the tumor microenvironment mediated by ascites [314,315,316]. The role of IF and IFP in cancer, as well as models of fluid shear stress in ovarian cancer, are discussed below [1,6,313].

### 3.1. Interstitial Fluid Pressure in Cancer

While direct evidence that ascites production is related to IF is lacking, computational and analytic models, along with indirect observations in patients and animal models, suggest that interstitial fluid transport is dysregulated in patients with ascites. In healthy tissue, IFP is tightly regulated to be within 3 mmHg of atmospheric pressure, but can be elevated as high as 100 mmHg in tumors [317]. Pressure gradients across blood vasculature, the interstitial space, and lymphatic vasculature drive IF, the flow of fluid through a 3D matrix and across the cells within the interstitial space [318,319]. As such, the leaky blood vessels, interstitial matrix contraction, and dysfunctional lymphatics commonly found in solid tumors lead to elevated IFP and IF [320]. Elevated IFP within the tumor decreases transport of nutrients and oxygen into the mass and generates higher rates of IF outward up to 55 μm/s in metastatic squamous cell cancer models, compared to rates of 0.1–2 μm/s in normal tissue [319,321]. As IF has been shown to drive morphogenesis, solute transport, and cell-cell signaling in healthy tissue, its significant dysregulation in cancer is of considerable interest [319].

Much of the previous work investigating IFP in cancer has examined its effects on the migration of breast cancer. IFP and it’s associated IF have been shown to influence the direction and propensity for migration through modulation of oxygen tension, upregulation of pro-metastatic signaling pathways, and ECM priming. Nathanson et al. [314] demonstrated that high IFP is associated with metastatic breast cancer in human patients. In these patients, metastatic tissue had an IFP of 5–52 mmHg compared to benign tumors with an IFP of 0.3–7.3 mmHg and healthy tissue with − 0.7–3.0 mmHg [314]. Later work by Rofstad et al. [316] found that elevated IFP within tumors caused higher IF rates, which have been shown to create an environment conducive to cellular invasion and directly impacts cell migratory potential. In response to IF, fibroblasts prime the ECM for cancer cell invasion through collagen degradation and elevated TGF-β1 activation [322]. Polacheck et al. [315] demonstrated that IF causes migration of MDA-MB-231 (human breast epithelial cell line) along the streamlines of IF, but there are competing C-C chemokine receptor type 7 (CCR7) dependent and independent mechanisms that determine whether cells migrate upstream or downstream. Overall, these publications demonstrate that IFP and its resulting IF are associated with metastatic tumors and influence the metastatic potential of cancer cells, but the mechanisms through which the forces are transduced to cellular responses are not fully understood.

Studies investigating the relationship between IFP and ovarian cancer have largely been performed in the context of drug delivery. To study the convective transport of large molecular weight compounds in ovarian cancer for tumor detection and drug delivery, Flessner et al. [323] developed a novel ovarian cancer model in rats by injecting ovarian cancer cells into the muscle of the abdominal wall to place tumors in discreet positions for tumor manipulation and in vivo measurements. Following tumor implantation, saline solutions were injected into the intraperitoneal space to raise the fluid pressure to 6–8 mmHg and induce a hydrostatic pressure gradient from the intraperitoneum into the tumor. Only moderate increases in tumor penetration were observed. The mean pressures within SK-OV-3 and NIH:OVCAR-3 tumors were found to be 9.7 ± 8.3 mmHg and 12.5± 7.0 mmHg, respectively. To induce convective transport into these tumors to aid in drug delivery, the pressure of the intraperitoneal space would have to be elevated to 12–15 mmHg, surpassing the pressure that can be sustained by the rat systemic circulation. After reducing tumoral pressure by administration of drugs or by decapsulation, drug penetration remained low. The authors concluded that in addition to the elevated tumor pressure, a physical component of the microenvironment, such as hyaluronan or collagen was impeding drug delivery to the tumor through electrostatic forces or bulk matrix density [324]. Subsequent work by the group tested these hypotheses by adding collagenase or hyaluronidase to the tumor and applying pressure to the intraperitoneal space. Tumors treated with hyaluronidase had a significant (>90%) decrease in hyaluronic acid within the tumor but maintained the same tumor IFP and IgG delivery before and after treatment, indicating that hyaluronic acid did not impact the hydraulic conductivity of IgG in the tumor. Tumors treated with collagenase did not have a dramatic decrease in collagen within the tumor but had a four-fold increase in penetration distance and had the tumor IFP drop from 13 mmHg to 9 mmHg. Elevated IFP due to ascites also imparts compressive forces on ovarian cancer aggregates embedded in the adjacent peritoneum. This compressive force has been demonstrated to exacerbate gene expression of E-cadherin and N-cadherin, which are associated with chemotherapy resistance and metastasis, respectively [325]. Overall, these studies demonstrated that both high IFP and the structure of the ECM within the tumor impede delivery of therapeutics to the ovarian cancer cells and directly impact their metastatic potential [326].

To study the distribution of IFP and IF within tumors and understand how controlling these variables could benefit patients, various models of IFP in tumors have been created. Jain et al. [327] suggested that normalizing vasculature could have multiple therapeutic impacts on tumors. By decreasing vascular permeability, less fluid leaks into the interstitial space, lowering the IFP, which can restore transvascular pressure gradients, increasing the transport of therapeutics into the tumor. The elevated IFP in tumors also pushed angiogenic factors towards the edge of the tumor and promoted lymphangiogenesis and metastasis. In order to model the impact of vessel permeability on IFP and IF in tumors, Jain et al. [327] used IFP measurements in a variety of human and animal models. 

Through these models, Jain et al. [327] demonstrated that the permeability of the vasculature has a significant impact on IFP and IF, and the transport of pro-metastatic factors can be minimized though normalization of the vasculature. Steuperaert et al. [328] created a similar model to study tumor pressure and drug delivery in vivo. Ovarian cancer cells suspended in Matrigel were injected into the subperitoneum of mice. After three weeks of tumor growth, the mice were subjected to 30 min of intraperitoneal chemotherapy treatment using cisplatin. A paramagnetic contrast agent was then injected intravenously for dynamic contrast-enhanced magnetic resonance imaging. It was found that IFP remained constant in the center of the tumor and dropped rapidly at the tumor margin, consistent with the findings of Jain et al. [327]. The model also matched experimental findings by Flessner et al., [323] where drug concentrations were highest at the edge of the tumor and significantly lower in the center of the tumor, possibly due to the higher IF at the tumor edge. Overall, studies on IFP in ovarian cancer have found that the elevated IFP within the tumor and the structure of the surrounding microenvironment impede drug delivery to the tumor [324,326,328], suggesting that IFP and IF can impact cell migration and chemotherapeutic efficacy in cancer models.

### 3.2. In Vitro Modeling of Fluid Stress in Ovarian Cancer

Here, we outline prior research dedicated to exploring shear stress-mediated effects on morphology and protein expression in ovarian cancer (summarized in Table 3). Overall, these studies have found that shear flow may induce EMT, increased motility, morphological changes, and chemoresistance.

Fluid shear stress is an important factor to consider in ovarian cancer, as it is known to affect proliferation, cytoskeletal remodeling, adhesion, and migration [287,288,332]. Since ascites-induced fluid shear stress values in ovarian cancer have not been precisely measured, approximations are based on gastrointestinal shear stress values (0.14–11 dyn/cm^2^) [287,333] and, although they may be physiologically relevant, quantification of shear stress values in malignant ascites in ovarian cancer is needed [313]. Experiments have shown that in the presence of fluid-flow induced wall shear stress (0.5–1.5 dyn/cm^2^), NIH:OVCAR-3 cells cultured in a monolayer displayed altered morphology, specifically elongation, as well as an increased stress fiber network compared to static cultures [287]. Cells exposed to flow-induced wall shear stress also demonstrated a densely organized network of cytoskeletal microtubules, indicating cytoskeletal remodeling in these cultures compared to static cultures [287]. Interestingly, there appeared to be a linear relationship between the magnitude of shear stress induced by the flow rate and the level of microtubule or stress fiber formation [287]. Based on the results from this study, Avraham-Chakim et al. [287] suggested that wall shear stress may induce cell motility, which is characterized by the presence of cell protrusions and contractile stress fibers, in ovarian cancer cells.

Flow-induced shear stress has also been shown to induce EMT, upregulate survival pathways and increase chemoresistance in 3D perfusion models for adherent ovarian cancer [9,10]. Compared to equivalent static cultures, 3D ovarian cancer nodules grown under continuous flow (0–3.3 dyn/cm^2^) showed an increase in hallmarks of EMT (e.g., loss of E-cadherin, increase in vimentin and increase in spindle-like morphology), as well as an increase in expression and activation of the EGFR [9]. It was also found that in the presence of flow, 3D ovarian cancer nodules exhibited decreased volume and viability, which is consistent with other findings that EMT is associated with decreased cell proliferation [51] and decreased viability of metastatic nodules resulting from migration-related stresses [56,334,335,336]. Overall, in this study, flow-induced shear stress was associated with an increase in EMT in 3D ovarian cancer nodules and upregulated biomarkers indicative of a more aggressive disease phenotype [9,337,338]. These findings were corroborated by Ip et al., [329] who also showed that flow-induced chemoresistance, specifically to cisplatin and paclitaxel, in non-adherent ovarian cancer spheroids. The finding that fluid shear stress induces resistance to cisplatin and paclitaxel is clinically relevant since drug resistance to platinum and taxane-based chemotherapy remains a barrier to effective treatment. Recently, in a perfusion model for adherent 3D ovarian cancer, Nath et al. [10] showed that flow-induced shear stress conferred resistance to carboplatin. A concomitant significant increase in EGFR expression and upregulation of MEK-ERK signaling was observed in adherent 3D ovarian cancer nodules grown under continuous flow and treated with carboplatin. Importantly, platinum concentration was significantly higher in 3D tumors grown under continuous flow, relative to corresponding static cultures, suggesting that, in this model, poor uptake of carboplatin under flow was not a major factor in the observed chemoresistance [10]. A mechanistically-distinct, photochemistry-based approach, photoimmunotherapy (PIT) was assessed to target 3D ovarian tumors grown under continuous flow. Based on the evidence of a flow-induced increase in EGFR expression, [9,10] as well the ability of PIT (and photodynamic therapy, PDT) to target tumors that do not respond to conventional therapies, [81,339,340,341,342,343,344,345,346,347,348,349] PIT was evaluated in 3D ovarian tumors grown under continuous flow. EGFR-targeted low-dose PIT was shown to be equally effective in 3D cultures grown under flow, compared to equivalent static conditions [10]. These findings, [9,10] among others, [81,339,340,341,342,343,344,345,346,347,348,349] highlight the need to consider physical stress in therapy design, and for further investigation into PIT/PDT-based priming of ovarian cancer to target chemoresistance due to flow-induced shear stress.

A study by Hyler et al. [288] utilized various ovarian cell lines, both tumorigenic and non-tumorigenic, to examine the effects of mechanotransduction from fluid stress on cell viability, organization, and genomic instability. Cells were placed on a rotator that continuously induced shear stress (0.13–0.32 dyn/cm^2^) on the cells through movement of the culture medium [288]. This method differs from those of the studies described above because rather than using a parallel flow system a rotating fluid flow system was employed, which may be more representative of peritoneal fluid motion [288]. In all cell lines tested, exposure to fluid shear stress without allowing cells to adhere resulted in a decrease in cell number compared to cells that were allowed to adhere [288]. Additionally, exposure of tumorigenic cells to fluid shear stress led to the formation of spheroids, with a highly aggressive mouse cell line forming larger, more aggressive spheroids, suggesting that increased exposure of spheroids to fluid shear stress can increase metastatic potential [288]. Additionally, actin protrusions, which are often associated with increased capacity for invasion and metastasis, were increased in tumorigenic cells exposed to fluid shear stress [288,350]. The presence of vinculin-containing focal adhesions was also increased in all cells under fluid shear stress, likely as a mechanism to increase cell adhesion [288]. Benign cells also exhibited chromosomal instability, specifically an increase in multi-lobed nuclei and tetraploidy, suggesting that fluid shear stress may induce malignant changes in benign cells [288]. Several models to apply fluid shear stress to cells have been developed and have shown altered molecular and morphological behaviors of ovarian cancer tumors and cells. Importantly, the observed effects of fluid shear stress are not limited to cancer cells. Both cytoskeleton and molecular profiling changes have been demonstrated in endothelial models; yet, the relationship between structure and signaling in all cell types requires further investigation.

### 3.3. In Vivo Modeling of Fluid Stress in Ovarian Cancer

Multiple studies have examined the relative suitability of ovarian cancer cell lines to model HGSOC in vivo. These studies have mainly focused on analyzing molecular profiles, the ability of cell lines to form tumors, and the formation of ascites. In this section, and in Table 4, we summarize the ovarian cancer cell lines that have been examined to model HGSOC in vivo. For the sake of focus, Table 4 lists the ovarian cancer cell lines that are most relevant to this review, prioritized, based on analysis of molecular profiles [351], to model HGSOC in vivo. Based on findings from Domcke et al. [351], the most suitable cell lines to model human HGSOC in vivo listed in Table 4 is the OVCAR-4 cell line followed by Caov-3, NIH:OVCAR-3, OV-90, OVCAR-8, HEY A8, SK-OV-3, A2780, and IGROV-1.

The in vivo suitability of these cell lines based on tumor take rate and ascites formation has also been studied. Hernandez et al. [352] reported on the ability of each cell line to form tumors in vivo based on microscopic tumor formation (ranked as low, medium, or high), and found that those with the highest capacity to form tumors are OVCAR-8, HEY A8, SK-OV-3, A2780, and ID8. Among the cell lines listed in Table 4, the NIH:OVCAR-3, OVCAR-4, OVCAR-5, OVCAR-8, and Caov-3 lines had the highest tumor take rates after intraperitoneal injection, as reported by Mitra et al. [353]. Some discrepancies in these results have been observed, however. Hernandez et al. [352] reported that NIH:OVCAR-3 cells show moderate ability to form disseminated disease post-intraperitoneal injection while Mitra et al. [353] reported a 6/6 tumor take rate for mice injected intraperitoneally with NIH:OVCAR-3 cells. Table 4 cites the relevant studies and lists the ability of NIH:OVCAR-3 cells to form tumors as medium-high. With respect to ascites formation in vivo, there are mixed results in the peer-reviewed literature. Several studies have shown that mice can develop ascites after intraperitoneal injection of NIH:OVCAR-3, OVCAR-8, HEY A8, SK-OV-3, A2780, OVCAR-5, and ID8 cells; however, there are contradictory studies with respect to the consistency of which these ascites form [352,353,354,355,356,357]. Due to inconsistencies in results across studies, the conclusions around ascites formation in vivo remain unclear. Some studies have found that NIH:OVCAR-3 cells frequently produce ascites in vivo while others have observed no ascites production [352,353,354,357]. Although comparative studies looking specifically at ascites volume in vivo after injection of a comprehensive panel of ovarian cancer cell lines have not been performed, the cell line that appears to form ascites in the most reproducible manner in vivo is SK-OV-3 [352,354,355].

Survival has also been examined in vivo after the injection of ovarian cancer cell lines. In the study by Mitra et al. [353], mice were sacrificed when tumor burden was evident, or the animals were moribund. If tumor growth was not evident by day 90, mice were sacrificed. After intraperitoneal injection of OVCAR-5, OVCAR-8, and NIH:OVCAR-3 cells, mice survived 26, 44, and 64 days, respectively. Mice injected with Caov-3 or OVCAR-4 cells were sacrificed on day 90. In another study looking at the ability of NIH:OVCAR-3 cells to produce ascites and intraperitoneal carcinomatosis in vivo, mice injected with 11.5 million cells survived on average 89 days while those injected with 40 million cells only survived an average of 38 days [357]. In this study, authors noted that once abdominal distension was observed due to ascites buildup and metastatic dissemination of the disease, mice could not survive more than 1–3 weeks. Other studies have also found that mice with extensive tumor burden have decreased survival compared to mice injected with slower growing or less aggressive cell lines [353]. Metastatic dissemination patterns in vivo that are similar to patients in the clinic have been reported for NIH:OVCAR-3, OVCAR-5, and SK-OV-3 cell lines [354,356,357]. Overall, survival of mice after injection of ovarian cancer cell lines appears to be associated with ascites formation and tumor dissemination. Further studies should explore whether there is a threshold for the relationship between ascites volume and survival of orthoptic mouse models of ovarian cancer.

**Table 4:** Ability of Ovarian Cancer Cell Lines to Form Tumors and Produce Ascites In Vivo.

Cell Line (i.p. Injection)	Species of Origin	Ability to Form Tumor	Ascites Present?
OVCAR-4	Human	Low-Medium [352,355]	No [352,353]
Caov-3	Human	Low [352]	No [352,353]
NIH:OVCAR-3	Human	Medium-High [352,355]	Yes [354,357] No [352,353]
OV-90	Human	Medium [352]	No [352]
OVCAR-8	Human	High [352]	Yes [353] No [352]
HEY A8	Human	High [352]	Yes [352]
SK-OV-3	Human	High [352,354]	Yes [352,354,355]
A2780	Human	High [352]	Yes [352]
IGROV-1	Human	Low [352]	No [352]
OVCAR-5	Human	Medium-High [352,356]	Yes [356] No [352,353]
ID8	Mouse	High [352]	Yes [352]

### 3.4. Compressive Force of Ascites

Interestingly, in addition to ascites-induced shear stress altering the tumor microenvironment, studies have suggested that the compressive force of ascites may contribute to tumor progression as well [358]. In advanced-stage or recurrent ovarian cancer, patients can present with >2 L of ascites, and this increased fluid volume in the peritoneum can drastically shift normal intraperitoneal pressure (IPP) from around 5 mmHg to as much as 22 mmHg [13,358,359]. This increase in intraperitoneal fluid volume and pressure can impose compressive forces within the peritoneal cavity that ultimately result in cell adhesion and metastasis [358]. In a murine model of ovarian cancer, Matsuzaki et al. [360] showed that increased IPP was associated with higher incidences of metastasis throughout the abdomen. Similarly, Asem et al. [358] found that in mice with artificial ascites, adhesion of OVCAR-5 and OVCAR-8 cells to peritoneal surfaces increased 20–30 fold. Furthermore, this study showed that compression of mesothelial cells led to altered cell morphology, acquisition of a mesenchymal phenotype, and formation of tunneling nanotubes, which can facilitate the cell-cell transfer of organelles under conditions of biophysical stress [358,361,362]. Overall, Asem et al. [358] concluded that, as a result of the alterations in the tumor microenvironment occurring under conditions of high IPP, ascites-induced compression can promote the adhesion, and metastatic potential, of ovarian cancer cells.

## 4. Treatment Options for Ascites

There is a paucity of treatment options for the management of malignant ascites [5,14,17,20,22,23]. Ascites fluid volume can be reduced through aspiration during debulking surgery or as a consequence of NACT. Patients presenting with malignant ascites may also undergo paracentesis; however, this technique only temporarily mitigates symptoms associated with malignant ascites and does not prevent recurrence. There are also significant complications associated with the treatment of ascites that need to be considered. While some pharmaceuticals have shown promise in clinical trials, severe complications and an overall lack of supporting evidence have limited their integration into the clinic. This section discusses treatment options for malignant ascites while highlighting the inadequacy of current therapeutic interventions and the need to focus on developing new treatments to meet the clinical challenges associated with malignant ascites.

Patients with intractable ascites often undergo frequent paracentesis, which involves the removal of ascitic fluid from the abdomen with a needle percutaneously. Becker et al. [17] reported that a mean of 94% of patients treated with paracentesis had temporary relief of symptoms when compiling data from five different cancer studies. Although this technique can temporarily relieve symptoms (e.g., ascites-related pain, swelling, and nausea), the risk of complications, such as draining site leakage or bowel perforation, must be considered [17,20,22]. To mitigate complications and improve success rates, ultrasounds can be used to assess the volume and location of intraperitoneal fluid as well as to confirm needle placement [363]. Although the use of ultrasound during paracentesis may reduce complication risk, paracentesis remains challenging, warranting the development of more effective strategies to treat malignant ascites.

While debulking surgery and paracentesis are most commonly used to decrease the volume of ascites in ovarian cancer patients, other techniques and pharmacological options have been explored. Some treatments, such as diuretics, have shown moderate success in the treatment of non-malignant ascites [364]; however, diuretics are contraindicated in the treatment of malignant ascites [17]. Catheters and peritoneovenous shunting have also been utilized in the treatment of malignant ascites, but these techniques are limited in clinical relevance, and are typically only used in end-stage patients. Several other pharmacological agents for the palliation of ascites have also been explored and are less commonly used clinically are discussed below.

Studies have shown that therapies such as bevacizumab, a VEGF-binding antibody, may not only delay the recurrence of platinum-sensitive [365] and platinum-resistant disease [366] if used with, or following, chemotherapy treatment, but may also palliate symptoms associated with ascites [20,367] Similarly, aflibercept, which binds VEGFA, VEGFB, and placental growth factor [368], may be able to decrease ascites accumulation and tumor proliferation through VEGF blockade [20,369,370]. Although trials using aflibercept in the palliation of ascites appear promising, a major complication of this therapy is bowel perforation, making its potential for therapeutic use in malignant ascites unclear [20,370]. Losartan is a Food and Drug Administration (FDA)-approved antihypertensive therapy that targets the renin-angiotensin system and has been evaluated to manage malignant ascites [23]. Zhao et al. [23] showed that in a mouse model of ovarian cancer, combination treatment of losartan and paclitaxel decreased tumor ECM content and subsequently decreased ascitic volume. In this study, decreased ECM content not only relieved lymphatic compression and improved drainage of ascites, but also enhanced the ability of injectable macromolecular drugs to infiltrate peritoneal tumors [23]. Losartan is currently not used in the management of ascites in patients with advanced-stage ovarian cancer, but may be of clinical relevance. Octreotide, a somatostatin analogue and VEGF inhibitor, is particularly useful for chylous ascites, which is a rare form of ascites containing large amounts of triglycerides; however, this treatment can reduce splanchnic blood flow, which can contribute to decreased lymph flow and bowel obstructive symptoms. The use of Octreotide for the treatment of malignant ascites has also been examined by Jatoi et al. [371] and revealed that monthly intramuscular injections of long-acting octreotide delayed the need for paracentesis in patients with malignant ascites from 14 to 28 days. Octreotide is typically not used in the management of patients with advanced-stage ovarian cancer since the data are limited and side effects remain a concern. Since MMPs have been implicated in increased tumor invasion and metastasis, targeting MMPs may be an effective strategy to manage malignant ascites. Batimatstat, an MMP inhibitor, decreased tumor growth, metastasis, and ascites volume in animal models [372,373,374,375,376]. Subsequently, it was administered to ovarian cancer patients intraperitoneally and was found only to decrease ascites volume in some patients [377,378]. This management strategy has not been widely adopted to manage ascites. Immunological agents have also showed promise in the treatment of malignant ascites. Studies have shown improvement in ascites volume with immunotherapy agents, such as intraperitoneal triamcinolone [379], intraperitoneal interferon α and β [380], tumor necrosis factor [381], and even non-pathogenic infectious agents [382,383,384]. Monoclonal antibodies have also been considered, and have shown success in mitigating ascites [385,386]. For example, catumaxomab is a trifunctional antibody (anti-CD3 + anti-EpCAM) that can kill tumor cells through immune-mediated mechanisms [20,22,387]. When undergoing Phase II/III trials in patients with malignant ascites, results showed that catumaxomab treatment led to longer puncture-free survival and fewer ascites-related symptoms [387]. While catumaxomab became the first therapeutic agent approved for the treatment of malignant ascites in Europe in 2009 [20,387], this modality has not been widely adopted in the United States.

PDT is a photochemistry-based therapeutic option that may be of value in the treatment of ovarian cancer and malignant ascites. Studies have shown that PDT, which in these studies refers to the combination of hematoporphyrin derivative and laser light, can effectively penetrate intraperitoneal tumors that are associated with the production of ascites and ascitic peritoneal spread in murine models [388,389]. Tochner et al. [388] revealed that when tumors were treated once with PDT, 90% of mice demonstrated decreased weight and abdominal size. Although this response was short-lived in mice receiving one round of treatment, 6 of 15 mice that received two rounds of PDT were alive at 90 days [388]. Another study by Tochner et al. [389] showed that 17 of 20 mice treated with four rounds of PDT were considered to be cured, and remained disease-free at 11 months. Mice treated only with either hematoporphyrin derivative or laser treatment continued to show tumor growth [389]. Based on results from these studies, PDT shows promise in the treatment of intraperitoneal tumors that are associated with the production of ascites and ascitic peritoneal spread. In the future, the effectiveness of PDT in the treatment of HGSOC and malignant ascites should be examined.

Another treatment option that may be used for the palliation of malignant ascites is the Sequana Medical Alfapump System, which is implanted subcutaneously and continuously drains intraperitoneal ascites via the urinary bladder [390]. The Alfapump was shown to decrease ascites volumes by 90% in patients with liver cirrhosis [391]. To examine the effectiveness and safety of the Alfapump in treating malignant ascites, Fotopoulou et al. [390] performed a retrospective study including 17 patients with recently implanted Alfapump systems with hepatic (35.3%) and ovarian (29.4%) malignancies being the most common. The median ascitic volume pumped daily was 303.6 mL, and the total median ascitic volume drained was 28 L [390]. Notably, the median number of paracenteses pre-implant was 4 compared to 1 post-implant [390]. This study showed the potential of the Alfapump system in treating malignant ascites and may warrant further investigation. 

Current methods used to treat malignant ascites in ovarian cancer lack both long-term effectiveness and desirability. Although some treatment options have shown moderate success in the palliation of ascites in the short-term, additional supporting data is crucial to determine the clinical feasibility and value of pharmacological agents and devices. Other techniques, such as PDT, initially examined in the treatment of ovarian cancer, should be examined further in the context of palliation of malignant ascites. In the future, it is critical that new agents or technologies are developed in order to prevent the accumulation and recurrence of malignant ascites in ovarian cancer.

## 5. Conclusions

Ovarian cancer is a highly heterogeneous disease in terms of genetic mutations and disease phenotypes and is divided into type I and type II tumors. Type II tumors, notably HGSOC, are more frequently associated with the production of malignant ascites (excess fluid containing malignant cells in the peritoneal cavity) and have increased genomic instability compared to type I tumors [25,29]. In addition to being associated with type II tumors, ascites production and volume are also correlated with advanced stage disease and increased metastatic spread [19,69]. Since ovarian cancer has the highest mortality rate of any gynecologic cancer, it is crucial to understand how malignant ascites contributes to disease progression in order to develop effective targeted therapies [1,392].

Malignant ascites comprises both acellular and cellular components that are involved in creating a tumor-promoting and immune-evading microenvironment. Acellular factors, such as integrins, can be activated via ascites fluid, and further advance cancer progression, invasion, metastasis, and immune disruption [125,130,131,133]. In addition, cytokines and growth factors, such as VEGF, IL-6, and IL-8, have been shown to promote angiogenesis, invasion, and even chemoresistance [88,89,90,91,104,105,106]. Interestingly, acellular factors upregulated in malignant ascites may induce EMT, which is indicative of more aggressive and disseminated disease [54,65,66,114,115]. Cellular factors present in malignant ascites that also play a role in these processes include components from both the innate and adaptive immune systems. Many of these factors (TAMs, NK cells, MSDCs, and T_regs_) are dysregulated in the ascites of ovarian cancer patients and are associated with immune suppression, chemoresistance, and decreased overall survival [190,202,217,218,232]. Ascites may also contribute to the dysregulation of lysosomal signaling, which has been linked to aggressiveness and chemoresistance in ovarian cancer. Altered lysosomal signaling and phenotypes can also lead to altered EV secretion. This is important because studies have reported that EVs play an important role in ovarian cancer progression and therapy response as well. While some standard EV isolation and separation techniques lack reproducibility, various methodologies, such as microfluidic platforms, have been reported for the robust isolation of EVs. This is critical since EVs may be useful for unveiling ovarian cancer biomarkers and tailoring precision medicine. To understand these cellular and acellular components of ascites, and their respective roles in ovarian tumor progression, metabolomic and proteomic profiling of patient-derived ascites have been performed [283,284,285,298].

Biophysical stresses imposed by ascites on the tumor may also contribute to disease progression. The magnitude and direction of ascitic flows in the peritoneum of supine patients is generally thought to be influenced by both intraperitoneal pressures and gravity [306,307], although more studies are needed in this area for a more precise understanding of how ascites impacts patients in the upright position. Other studies have confirmed the importance of interstitial pressure and interstitial fluid flow in tumor progression and therapy response in a variety of cancer models [314,315,316,324,326,328]. In order to model these intraperitoneal flows, 3D perfusion models and microfluidic platforms have been utilized, and studies have found that fluid flow may induce EMT and chemoresistance in ovarian cancer cells [9,10,329].

Malignant ascites and the associated tumor-promoting microenvironment can alter therapy response. As a result, the presence of malignant ascites in advanced-stage ovarian cancer patients is associated with a poor prognosis, warranting the development of more effective treatments for the palliation of malignant ascites. Commonly, ascites volume can be reduced through aspiration during debulking surgery or as a consequence of NACT. As for patients with intractable ascites, paracentesis is frequently utilized; however, there may be serious complications [17,20]. Additionally, other techniques, including PDT, initially explored for the treatment of ovarian cancer [388,389,393], have shown effectiveness in decreasing ascites volume and therefore warrant further examination. Pharmacological treatments such as VEGF inhibitors, MMP inhibitors, and immunological agents have also been examined, and while supporting data are limited, they have demonstrated promising outcomes [365,366,369,372,373,374,375,376,379,380,381,382,383,384,385,386,387].

Malignant ascites is frequently found in patients with advanced-stage and metastatic ovarian cancer and contributes to tumor proliferation, invasion and metastasis through various cellular, acellular, and biophysical mechanisms, hampering the effectiveness of conventional therapies. Current therapies used in the management of ascites can be useful initially; however, many of these therapies lack long-term efficacy. Exploratory therapeutic options such as VEGF and MMP inhibitors have demonstrated clinical success; however, studies are limited. As a result, the development of effective therapeutic options for the treatment of malignant ascites is needed and understanding the tumorigenic role of malignant ascites is a crucial step in achieving this goal.

## Figures and Tables

**Table 1:** Cytokines and Growth Factors in Malignant Ascites.

Cytokine/Growth Factor	Role
Cancer Antigen 125 (CA-125)	Promotes ovarian tumor growth and metastasis [78] Protects ovarian tumor cells from recognition by NK cells [79]
Epidermal Growth Factor (EGF)	Regulates proliferation, growth, and survival [80,81,82] Associated with aggressive, invasive, and metastatic phenotype for ovarian cancer [83,84,85] Downregulates E-cadherin, which is indicative of EMT [61]
Interleukin-6 (IL-6)	Promotes ascites formation and ovarian cancer progression [86] Associated with shorter progression-free survival [86,87] Promotes migration, invasion [88], chemoresistance [89], and angiogenesis [90]
Interleukin-8 (IL-8)	Promotes tumor proliferation, adhesion, invasion, and angiogenesis [91] Increased tumorigenicity and ascites formation in animal models [92]
Interleukin-10 (IL-10)	Associated with shorter progression-free survival [93] Confers resistance to tumor necrosis factor-related apoptosis-inducing ligand (TRAIL)-induced apoptosis [93] Assists in evasion of immunological surveillance [94,95,96]
Lysophosphatidic Acid (LPA)	Regulates transcription of VEGF, uPA, IL-6, and IL-8 [72,97] Disrupts integrity of ovarian cancer cells, facilitating dissemination and metastasis [98]
Urokinase Plasminogen Activator (uPA)	Involved in ECM organization [99], cell-ECM adhesion [100], and cell motility [101] Associated with proliferation, migration, invasion, and advanced-stage disease [102,103]
Vascular Endothelial Growth Factor (VEGF)	Enhances tumor growth, invasion, and metastasis [104] Assists in ascites formation and cancer progression [105,106] Downregulates tight junction protein Claudin-5 [107]

**Table 3:** Molecular Alterations of Ovarian Cancer Cells Subject to Flow-Induced Shear Stress.

Reference	Major Findings
Avraham-Chakim et al., 2013. [287]	Cells under shear stress had increased elongation, increased stress fiber network, and a more densely organized network of cytoskeletal microtubules Magnitude of shear stress positively related to microtubule formation
Rizvi et al., 2013. [9]	Compared static and flow cultures Upregulated EGFR expression and p27Kip1 under flow Decreased E-cadherin protein expression and increase in vimentin under flow
Ip et al., 2016. [329]	Phospho (p)-Akt, Akt, p-p70^S6K^ and p70^S6K^ proteins upregulated under fluid shear stress
Hyler et al., 2018. [288]	Tumorigenic cells formed larger and more aggressive spheroids and more actin protrusions compared to benign cells Fluid shear stress increased vinculin-containing focal adhesion assembly Benign cells exhibited chromosomal instability under shear flow
Li et al., 2019. [330]	Metastatic cancer stem cells experienced sialyl-Lewisx-P-selectin mediated binding under fluid shear stress
Sun et al., 2019. [331]	Fluid flow induced IL-8 protein production
Nath et al., 2020. [10]	Compared static and flow cultures Phosphorylated-ERK1/2 increased under flow Phospho-paxillin and phospho-FAK and vinculin decreased under flow

## Data Availability

Not applicable.

## Abbreviations

AA: Arachidonic acid; ACC, Acetyl-CoA carboxylase; ADCC, Antibody-dependent cellular cytotoxicity; AKT, Protein Kinase B; ARID1A, AT-rich interaction domain 1A; ATP, Adenosine triphosphate; Bcl-xL, B-cell lymphoma-extra-large; BDCA, Blood dendritic cell antigen; BLT2, Leukotriene B4 receptor 2; BRAF, B-Raf proto-oncogene, serine/threonine kinase; BRCA1/2, Breast cancer gene 1/2; B7-H4, Immunoreceptor family B7, receptor H4; B7-H6, Immunoreceptor family B7, receptor H6; c-Myc, Cellular myelocytomatosis; CA-125, Cancer antigen-125; CAF, Cancer-associated fibroblast; CCL, Chemokine ligand; CCNE1, Cyclin E1; CCR7, C-C chemokine receptor type 7; CD, Cluster of differentiation; COX, Cyclooxygenases; CSF-1, Colony stimulating factor-1; CT, Computed tomography; CTNNB1, Catenin β1; CXCL, Chemokine (C-X-C motif) ligand; Cyr61, Cysteine-rich protein 61; DC, Dendritic cell; DNA, Deoxyribonucleic acid; ECM, Extracellular matrix; EGF, Epidermal growth factor; EGFR, Epidermal growth factor receptor; EMT, Epithelial-mesenchymal transition; EOC, Epithelial ovarian cancer; EpCAM, Epithelial cell adhesion molecule; EP1–4, Prostaglandin E2 receptors 1–4; ERK, Extracellular signal-related kinase; EV, Extracellular vesicle; FAK, Focal adhesion kinase; FAP, Fibroblast activation protein; FDA, Food and Drug Administration; FDT1, Squalene synthase; FIGO, International Federation of Gynecology and Obstetrics; Flk-1, Vascular endothelial growth factor receptor 2; FOXM1, Forkhead box M1; FOXP3, Forkhead box protein 3; GARP, Glycoprotein A repetitions predominant; GC-MS, Gas chromatography-mass spectrometry; GDLC, Glycine dehydrogenase; GM-CSF, Granulocyte-monocyte colony stimulating factor; GRO-1, Growth-related oncogene-1; HB-EGF, Heparin-binding epidermal growth factor-like growth factor; HETE, Hydroxyeicosatetraenoic acid; HGF, Hepatocyte growth factor; HGSOC, High-grade serous ovarian carcinoma; HIF-1α, Hypoxia-inducible factor 1-alpha; IDO, Indoleamine 2,3-dioxygenase; IF, Interstitial fluid flow; IFN-γ, Interferon gamma; IFP, Interstitial fluid pressure; Ig, Immunoglobulin; IGF-1, Insulin-like growth factor-1; IL, Interleukin; ILC, Innate lymphoid cell; iNOS, Inducible nitric oxide synthase; IPP, Intraperitoneal pressure; JAK 3, Janus Kinase 3; KRAS, Kirsten rat sarcoma viral oncogene homolog; LAMP1, Lysosome-associated membrane protein-1; LDH, Lactate dehydrogenase; LIF, Leukemia inhibitory factor; LOX, Lipoxygenases; LPA, Lysophosphatidic acid; LTB4, Leukotriene B4; MAP, Mitogen-activated protein; MAPK, Mitogen-activated protein kinase; MC, Mesothelial cells; MDSCs, Myeloid-derived suppressor cells; MEK, Mitogen-activated protein kinase/extracellular signal-related kinase; MET, Mesenchymal-epithelial transition; MHC, Major histocompatibility complex; miRNA, Micro ribonucleic acid; MMP, Matrix metalloproteinase; mRNA, Messenger ribonucleic acid; MRP4, Multidrug resistance protein 4; mTORC1, Rapamycin complex 1; NACT, Neoadjuvant chemotherapy; NF1, Neurofibromin-1; NK cell, Natural killer cell; NLR, Neutrophil to lymphocyte ratio; Oct4, Octamer-binding transcription factor 4; OSE, Ovarian surface epithelium; PD-1, Programmed cell death protein 1; PDGF, Platelet-derived growth factor; PD-L1, Programmed death-ligand 1; PDT, Photodynamic therapy; PG, Prostaglandin; PI3K, Phosphoinositide 3-kinase; PIK3CA, Phosphatidylinositol-4,5-bisphosphate 3-kinase catalytic subunit α; PIT, Photoimmunotherapy; PLA_2_G7, Phospholipase A2 group VII; PTEN, Phosphatase and tensin homolog; q-PCR, Quantitative polymerase chain reaction; RAS, Rat sarcoma; RB, Retinoblastoma protein; RNA, Ribonucleic acid; SDS-PAGE, Sodium dodecyl sulfate-polyacrylamide gel electrophoresis; SID-SRM, Stable isotope dilution-selected reaction monitoring; S1P, Sphingosine-1-phosphate; SRM-MS, Selected reaction monitoring-mass spectrometry; STAT3, Signal transducer and activator of transcription 3; TAM, Tumor-associated macrophage; T_c_, Cytotoxic T cell; TGF, Transforming growth factor; T_h_, Helper T cell; Th17, T helper 17 cell; TICs, Tumor-initiating cells; TNF, Tumor Necrosis Factor; TP53, Tumor protein 53; TRAIL, Tumor necrosis factor-related apoptosis-inducing ligand; T_reg_, Regulatory T cell; TXA_2_, Thromboxane A2; TXB_2_, Thromboxane B2; uPA, Urokinase plasminogen activator; uPAR, Urokinase plasminogen activator receptor; UPR, Unfolded protein response; VCAM-1, Vascular cell adhesion molecule 1; VEGF, Vascular endothelial growth factor; Wnt, Wingless-related integration site; XBP1, X-box binding protein 1; α-SMA, Alpha-smooth muscle actin.
